# PRDM16 Drives Thyroid Cancer Differentiation via a TRIM58-MVP Axis to Suppress MAPK and PI3K/AKT Signaling

**DOI:** 10.7150/ijbs.131151

**Published:** 2026-04-23

**Authors:** Jialong Yu, Wei Luo, Zijie Niu, Miao Zhang, Yunqi Gao, Mengran Tian, Zhongyu Wang, Xing Wan, Han Gui, Qian Su, Wenhao Chen, Linfei Hu, Xiukun Hou, Qiman Dong, Xianhui Ruan, Xiangqian Zheng

**Affiliations:** 1 Department of Thyroid and Neck Tumor, Tianjin Medical University Cancer Institute and Hospital, Tianjin's Clinical Research Center for Cancer, Tianjin, China.; 2 School of Medicine, Nankai University, Tianjin, China.; 3 Department of Thyroid and Breast Surgery, Tianjin Union Medical Center, The First Affiliated Hospital of Nankai University, Tianjin, China.; 4 State Key Laboratory of Advanced Medical Materials and Devices, Tianjin Key Laboratory of Radiation Medicine and Molecular Nuclear Medicine, Key Laboratory of Radiopharmacokinetics for Innovative Drugs, Tianjin Institutes of Health Science, Institute of Radiation Medicine, Chinese Academy of Medical Sciences & Peking Union Medical College, Tianjin, China.; 5 Department of Molecular Imaging and Nuclear Medicine, Tianjin Medical University Cancer Institute and Hospital, Tianjin's Clinical Research Center for Cancer, Tianjin, China.

**Keywords:** PRDM16, thyroid cancer, differentiation, TRIM58, MVP

## Abstract

Thyroid cancer frequently undergoes dedifferentiation, progressing into poorly differentiated or anaplastic carcinoma, which is characterized by therapeutic resistance and a poor prognosis. Epigenetic dysregulation, particularly histone methylation, plays a critical role in this process. In this study, we identify PRDM16 as an essential regulator of thyroid cancer differentiation. Mechanistically, we demonstrate that PRDM16 catalyzes H3K9 monomethylation at the TRIM58 promoter region, thereby enhancing TRIM58 transcription. Upregulated TRIM58 subsequently promotes ubiquitination and degradation of MVP, leading to suppression of both the MAPK and PI3K/AKT signaling pathways and maintenance of cellular differentiation. This PRDM16-TRIM58-MVP axis modulates proliferation, epithelial-mesenchymal transition, and radioiodine uptake in thyroid cancer cells. Moreover, PRDM16 overexpression enhances the efficacy of MAPK inhibitor-induced redifferentiation therapy. Collectively, these findings establish PRDM16 as a novel tumor suppressor and potential therapeutic target, offering a promising redifferentiation-based strategy for the treatment of advanced thyroid cancer.

## Introduction

Thyroid cancer is the most common endocrine malignancy, and its incidence has been steadily increasing worldwide over recent decades 1,2. Most cases are well-differentiated subtypes, such as papillary thyroid carcinoma (PTC) and follicular thyroid carcinoma (FTC), which generally have favorable prognoses owing to their responsiveness to conventional treatments, including surgical resection, radioactive iodine (RAI) ablation, and thyroid-stimulating hormone (TSH) suppression therapy 3. However, a subset of tumors undergoes dedifferentiation, a process marked by the loss of thyroid-specific functions and morphological features, leading to poorly differentiated thyroid carcinoma (PDTC) or anaplastic thyroid carcinoma (ATC). These aggressive forms are highly lethal, exhibit a poor response to standard therapies, and are associated with dismal outcomes 4,5.

Dedifferentiation represents a critical transition in thyroid cancer, characterized by therapeutic resistance and aggressive clinical behavior. During this process, tumor cells progressively downregulate thyroid hormone biosynthesis and developmental regulators, including NIS, PAX8, and NKX2-1, thereby contributing to RAI refractoriness and chemotherapy resistance 6,7. Redifferentiation therapy first achieved remarkable advances in the treatment of acute promyelocytic leukemia, a disease now curable through the combination of retinoic acid and arsenic 8,9. Furthermore, redifferentiation therapy has also been explored in various solid tumors, retinoic acid has been demonstrated to reactivate NF-κB kinase subunit IKKA, promoting differentiation in nasopharyngeal carcinoma and neuroblastoma cells 10,11. Therefore, by promoting the redifferentiation of highly malignant, poorly differentiated cancer cells, this approach has emerged as a promising therapeutic avenue. In thyroid cancer, aberrant activation of the MAPK and PI3K/AKT signaling pathways contributes to tumor proliferation and dedifferentiation, at least in part by repressing iodide-handling genes such as NIS and downregulating thyroid-specific transcription factors, including PAX8 12-15. Targeted inhibition of the MAPK pathway, particularly through MEK inhibitors, has been shown to partially restore NIS expression and resensitize radioiodine-refractory thyroid cancer to RAI therapy, highlighting redifferentiation therapy as a potential breakthrough for advanced disease 16-18. Despite these advances, not all patients with radioiodine-refractory thyroid cancer respond to BRAF inhibition or exhibit evidence of redifferentiation 19,20, suggesting that additional epigenetic mechanisms cooperate with signaling pathways to determine the differentiation state of thyroid cancer cells.

Epigenetic modifications play pivotal roles in regulating gene expression and cell fate decisions. Among epigenetic regulators, histone methylation plays a pivotal role in tumorigenesis and tumor progression 21. By adding methyl groups to lysine or arginine residues on histones, histone methylation alters chromatin architecture and gene expression 22. Disruption of the balance between histone methylation and demethylation contributes to oncogenesis 23,24. Increasing evidence shows that histone methylation-related genes regulate differentiation in various cancers. For example, KDM2B enrichment in pancreatic cancer correlates with tumor grade and poor differentiation, while its silencing abolishes tumorigenicity in poorly differentiated cells 25. Similarly, KDM5B expression in leukemia promotes differentiation by suppressing stemness-associated transcriptional programs 26. Our previous work revealed that the histone methyltransferase SETMAR, identified as the top-ranked candidate in a correlation analysis between histone methyltransferase expression and the Thyroid Differentiation Score (TDS) in TCGA patients, promotes thyroid cancer differentiation via SMARCA2-mediated chromatin remodeling 27. The second-ranked candidate, PRDM16, however, remains uncharacterized. Here, we aimed to elucidate the role of PRDM16 in thyroid cancer differentiation.

PRDM16, a member of the PR-domain-containing protein family, harbors a conserved N-terminal PR domain and multiple zinc finger motifs 28. Its PR domain shares homology with the SET domain, a characteristic of histone methyltransferases. PRDM16 possesses intrinsic histone methyltransferase activity, catalyzing H3K9 monomethylation (H3K9me1) 29. Beyond its chromatin-modifying activity, PRDM16 can function as a transcriptional regulator by forming complexes with histone-modifying enzymes. Notably, PRDM16 has been reported to act as a tumor suppressor in several malignancies 30-32.

In this study, we demonstrate for the first time that PRDM16 acts as a histone methyltransferase to maintain thyroid cancer cell differentiation. PRDM16 catalyzes H3K9me1 modification, thereby promoting the transcriptional activation of TRIM58. As an E3 ubiquitin ligase, TRIM58 mediates the ubiquitination and degradation of MVP, leading to suppression of both MAPK and PI3K/AKT signaling pathways. This PRDM16-TRIM58-MVP axis regulates differentiation, proliferation, epithelial-mesenchymal transition (EMT), and RAI uptake. Furthermore, PRDM16 overexpression enhances sensitivity to MAPK inhibitor-induced redifferentiation therapy. These findings establish PRDM16 as a novel tumor suppressor and a promising therapeutic target, highlighting its potential in developing new redifferentiation strategies for advanced thyroid cancer.

## Materials and Methods

### Clinical Data and Tissue Samples

The study involved tumor tissue sections from 113 patients with complete medical records at Tianjin Medical University Cancer Institute and Hospital, including diagnosed cases of PTC, PDTC, and ATC. Matched fresh PTC tissues and adjacent normal thyroid tissues were obtained from 26 patients. Total RNA was isolated from 18 paired samples, and total protein was extracted from 8 paired samples. This study was approved by the Ethics Committee of Tianjin Medical University Cancer Institute and Hospital (Approval ID: bc20251044), and informed consent was obtained for the experimental use of human tissues.

### Database and Data Analysis

Transcriptome data and clinical information of THCA patients were obtained from The Cancer Genome Atlas (TCGA-THCA) database. RNA-seq expression data were normalized to transcripts per million (TPM) values through log2(x+1) transformation. GES33630, GSE29265, and GSE76039 were obtained from the Gene Expression Omnibus (GEO). Gene expression and genomic data were obtained from the Cancer Cell Line Encyclopedia (CCLE) database. The batch effects across datasets were adjusted using the removeBatchEffect function from the limma package (version 3.54.0) in R 4.1.1, followed by integration of the harmonized datasets. Single-cell RNA sequencing (scRNA-seq) data were analyzed from the GSE193581, GSE250521, GSE148673, and GSE182416 datasets. The Markov Affinity-based Graph Imputation of Cells (MAGIC) algorithm was applied to correct the "drop-out" phenomenon commonly observed in scRNA-seq data 33. The Harmony algorithm was then used to integrate out the batch effects. Subsequently, 3,000 hypervariable genes were selected for principal component analysis (PCA) to reduce dimensionality. The SNN algorithm was used for clustering and the t-SNE algorithm is used for visualization. The AddModuleScore algorithm was used to visualize cell classification based on TDS scores.

### Total RNA Extraction and Quantitative Real-time PCR

Total RNA was extracted from thyroid cancer cell lines and fresh clinical tissues using RNA Extraction Reagent (G3013; Servicebio, China). Complementary DNA (cDNA) was synthesized from mRNA using the HiScript II 1st Strand cDNA Synthesis Kit (R212-01; Vazyme, China). Quantitative real-time PCR (qRT-PCR) was performed with HiScript II Q RT SuperMix (R223-01; Vazyme, China) and gene-specific primers following the manufacturer's instructions. Relative mRNA expression levels were calculated using the 2-^ΔΔCt^ method with β-actin as the internal control for normalization. Primer sequences are provided in Additional File 1.1.

### Immunohistochemistry (IHC)

Clinical tumor tissue and xenograft tumor tissue were fixed in 10% neutral buffered formalin and then embedded in paraffin. Sections were stained with the indicated antibodies according to the immunohistochemistry protocol. Signals were visualized using a DAB Substrate Kit (ZLI-9017; ZSBIO, China). Histochemical scoring was independently performed by two pathologists using the following methodology: Histoscore = Staining intensity × Percentage of positive tumor cells. Staining intensity was graded as 0 (no staining), 1 (weak, light yellow), 2 (moderate, light brown), or 3 (intense, brown). The percentage of positive cells was categorized as 0 (<5%), 1 (5-25%), 2 (26-50%), 3 (51-75%), or 4 (> 75%).

### RNA-Seq

PRDM16-overexpressing or control CAL-62 cells were lysed using an RNA extraction solution (G3013; Servicebio, China) and the mRNA was extracted according to the manufacturer's protocol. The global gene expression profiles were determined by mRNA sequencing, which was performed at TIANGEN Biotechnology (Beijing, China) Co., Ltd.

### Chromatin Immunoprecipitation (ChIP)

For each ChIP sample, 1 × 10^6^ cells were fixed with 1% formaldehyde, and the cross-linked chromatin was sonicated to generate 200-800 bp DNA fragments for subsequent immunoprecipitation. The protein/DNA complexes were then immunoprecipitated, separated, and purified. Purified DNA was used for qPCR analysis or to generate a second-generation sequencing library, which was subsequently sequenced on a NovaSeq PE150 by Novogene (Beijing, China). The sequences of the primers that were used for ChIP-qPCR are listed in Additional file 1.2.

### Cell Transfection and Lentiviruses Infection

The PRDM16 and PRDM16-ΔPRD overexpression lentiviruses were purchased from GENECHEM Co., Ltd. (Shanghai, China), and the GV492 vector was used as a negative control. Small interfering RNAs (siRNAs), the overexpression plasmid TRIM58, pcDNA3.1-MVP-Myc, MVP truncation mutants, and MVP ubiquitin mutants were all purchased from Hanbio Biotechnology (Shanghai, China) Co., Ltd.‌ The sequences of siRNAs are listed in Additional File 1.3. siRNAs or plasmids were transfected into cells using Lipofectamine 3000 (L3000075, Thermo Fisher Scientific, USA). For lentiviral infection, cancer cells were selected in the presence of 1 μg/mL puromycin to establish stable cell lines.

### *In Vitro* ^131^I Uptake Assay

Thyroid cancer cells (5 × 10^5^ cells/well) were seeded in triplicate into 12-well plates. Cells were incubated in serum-free medium supplemented with1 μCi Na^131^I and 10 μM NaI at 37°C for 30 minutes. For the control group, cells were pretreated with the competitive NIS inhibitor NaClO_4_ (300 μM) for 30 minutes prior to Na^131^I exposure to assess non-specific radioactive iodine uptake. Following treatment, cells were washed twice with ice-cold PBS and lysed with 0.5 mL of 0.3 M NaOH. Radioactivity levels were quantified using a PerkinElmer 2470 gamma counter.

### *In Vivo* ^131^I Uptake Assay

Prior to Na^131^I uptake assays mice received drinking water supplemented with 0.05% potassium iodide (Sangon, A100512) for 7 d. *In vivo* imaging was performed using a multi-modal preclinical PET/SPECT/CT system (InliView-3000B, Novel Medical, China). Each animal was intravenously injected with 300 μCi of Na¹³¹I via the tail vein, and SPECT acquisitions were initiated 10 min post-injection. Reconstruction and export of the datasets were performed in NMSoft-AIWS (Novel Medical). Radioactivity in subcutaneous tumors and thyroids was measured as counts per minute using a gamma counter.

### Immunoprecipitation (IP) and Co-immunoprecipitation (Co-IP)

Cell samples were lysed with 1% NP-40 buffer (50 mM Tris-HCl pH 7.4, 150 mM NaCl, 1% NP-40), which was supplemented with a protease inhibitor cocktail (Solarbio, China) at 4 °C for 30 min. The cell lysates were subjected to overnight incubation at 4 °C with primary antibodies, followed by a 4 h incubation at 4 °C with Protein A/G-agarose beads (Beyotime, China). Immunoprecipitates were subsequently washed with 0.05% NP-40 buffer and detected by western blotting analysis.

### Mass Spectrometry Analysis

The cell lysate was obtained as described in the aforementioned IP steps. Flag magnetic beads (ZC387750, Thermo Fisher Scientific, USA) were then added to the lysate to isolate the IP products. The IP products were separated by SDS-PAGE. The gel was subsequently stained with a commercial silver staining kit (Beyotime, China) according to the manufacturer's instructions to verify the success of immunoprecipitation. For mass spectrometry (MS) analysis, parallel IP products were resolved by SDS-PAGE and stained with Coomassie Brilliant Blue. Gel regions without visible protein bands were excised and processed as background controls. Mass spectrometric analysis was performed by Beijing Qinglian Biotechnology Co., Ltd.

### Antibodies and Drugs

The antibodies used in this study are listed in Additional file 1.4. Cycloheximide (CHX), MG132 and Selumetinib were all purchased from Selleck Chemicals (Houston, TX, USA).

### Animal Studies

Six-week-old female BALB/c nude mice were purchased from SPF Biotechnology Co., Ltd (Beijing, China) and maintained under specific pathogen-free conditions. Tumor xenograft models were established by subcutaneously injecting 2 × 10^6^ cells into the flanks of mice. Tumor growth was monitored every 5-7 d after tumors reached an average volume of 50 mm^3^, and tumor volume was calculated using the formula: volume = length × width^2^ / 2. For experiments without drug intervention, mice were monitored until the indicated endpoints. For drug treatment experiments, when tumors reached approximately 50 mm^3^, mice were treated with selumetinib (10 mg/kg) once daily by intragastric gavage, as described previously 27, while control mice received vehicle following the same schedule.

### Statistical Analysis

All data were analyzed using GraphPad Prism 9.0.0 software. Each experiment was conducted at least 3 times and the data were presented as mean ± standard deviation (SD). Kaplan-Meier analysis was used to evaluate survival curves, and the differences in the survival probabilities were assessed using the log-rank test. Paired Student's t-test was performed to analyze matched PTC and corresponding normal thyroid tissue samples. Pearson correlation analysis was employed to assess correlations between gene expression levels. The two-tailed Student's t-test was used to compare two independent groups, and one-way or two-way ANOVA was used for multi-group comparisons. Statistical significance was defined as *P* < 0.05 (***P < 0.001, **P < 0.01, *P < 0.05).

## Results

### PRDM16 is a Key Regulatory Factor in Thyroid Cancer Differentiation

**Figure 1:**
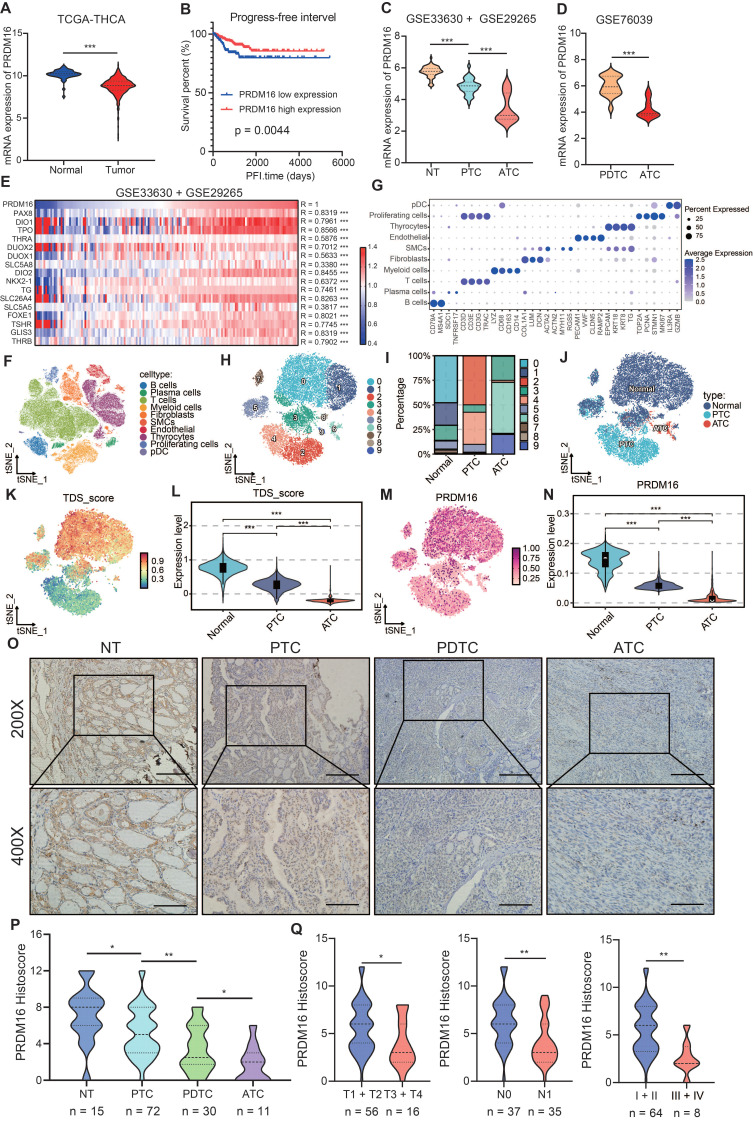
** PRDM16 plays a critical role in promoting the differentiation of thyroid cancer.** (A) Analysis of PRDM16 mRNA expression in PTC and normal thyroid tissues in the TCGA dataset. (B) Kaplan-Meier analysis of PFS according to PRDM16 expression in the TCGA dataset. (C, D) Analysis of PRDM16 expression in different types of thyroid carcinoma and normal thyroid tissue in the GSE33630 and GSE29265 (C), and GSE76039 (D) databases. (E) Heatmap showing the correlation between PRDM16 and TDS-related genes in the GSE33630 and GSE29265 databases. (F-H) Single-cell RNA-seq analysis of thyroid samples: including t-SNE clustering of thyroid cells (F), marker gene expression across clusters (G), and distribution of major cell types(H). (I) Proportions of thyroid follicular and tumor cell subsets in NT, PTC, and ATC samples. (J) t-SNE visualization comparing thyroid follicular cells and tumor cells. (K, L) Clustering of thyroid follicular and tumor cells validated by TDS differences. (M, N) Comparison of PRDM16 expression among thyroid follicular cells, PTC cells, and ATC cells. (O) IHC staining of PRDM16 in normal thyroid (NT) tissue, PTC tissue, PDTC tissue, and ATC tissue. (P) Quantification of PRDM16 expression in different thyroid cancer subtypes. (Q) Correlation of PRDM16 immunohistochemical staining with tumor size, lymph node metastasis, and clinical stage in PTC patients. All data represent mean ± SD. Statistical significance was determined by unpaired Student's t-test (A, C, D, L, N, P, Q).

To evaluate the expression of PRDM16 in thyroid cancer patients, we first analyzed the gene expression dataset of thyroid cancer. According to the analysis of the TCGA database, we found that PRDM16 is lowly expressed in thyroid cancer compared to normal tissues and leads to poorer Progression Free Survival (PFS) (Figure 1A, B). Through the analysis of GEO datasets including GSE33630, GSE29265, and GSE76039, we found that PRDM16 expression shows a declining trend in thyroid cancer with reduced differentiation (Figure 1C, D). Based on the analysis of TCGA and GEO datasets, we observed a significant positive correlation between PRDM16 expression and most TDS-related genes (Figure 1E, S1A), as well as a direct positive association with the TDS score (Figure S1B). Analysis of publicly available CCLE data showed that PRDM16 mRNA expression was significantly higher in well-differentiated thyroid cancer cell lines than in poorly differentiated and anaplastic thyroid cancer cell lines (Figure S1C). Moreover, PRDM16 expression levels were significantly associated with various clinical characteristics of thyroid cancer, including T stage, N stage, and clinical stage (Figure S1D).

Subsequently, we analyzed single-cell RNA sequencing datasets to investigate the impact of PRDM16 expression patterns on thyroid cancer differentiation at the single-cell level. We integrated single-cell RNA sequencing data from multiple samples in the GSE148673 and GSE184362 datasets, including normal thyroid tissues, PTC, and ATC. Through data integration, dimensionality reduction, and clustering analysis, we classified distinct cell populations (Figure 1F-J). Furthermore, we specifically isolated normal thyroid follicular cells, PTC cells, and ATC cells to determine the differences in TDS level and PRDM16 expression levels among these three cell types (Figure 1K, L). The results indicated that the TDS level and PRDM16 expression were lower in tumor cells, particularly in ATC cells (Figure 1M, N).

Finally, we validated the expression of PRDM16 in the collected clinical samples. We extracted and analyzed RNA and proteins from paired PTC samples and adjacent normal tissues. The results demonstrated significantly lower PRDM16 expression in PTC compared to normal tissues (Figure S1 E-G). Next, we examined PRDM16 protein expression using IHC staining in normal thyroid tissues (n = 15), PTC, (n = 72), PDTC (n = 30), and ATC (n = 11) (Figure 1O). The results demonstrated that PRDM16 was downregulated in tumor tissues, with its expression level progressively decreasing as tumor differentiation declined (Figure 1P). In PTC samples, PRDM16 expression was significantly associated with clinicopathological features. Its levels decreased with advancing T stage, were lower in tumors with lymph node metastasis (N1) than in those without (N0), and were markedly reduced in advanced clinical stages (III-IV) compared with early stages (I-II) (Figure 1Q). Furthermore, we analyzed PRDM16 expression in human normal thyroid, PTC, and ATC cell lines. Compared with normal thyroid cell, PRDM16 was downregulated in thyroid cancer cells, particularly in ATC cells (Figure S1 H, I).

### PRDM16 Facilitates Thyroid Cancer Differentiation

**Figure 2:**
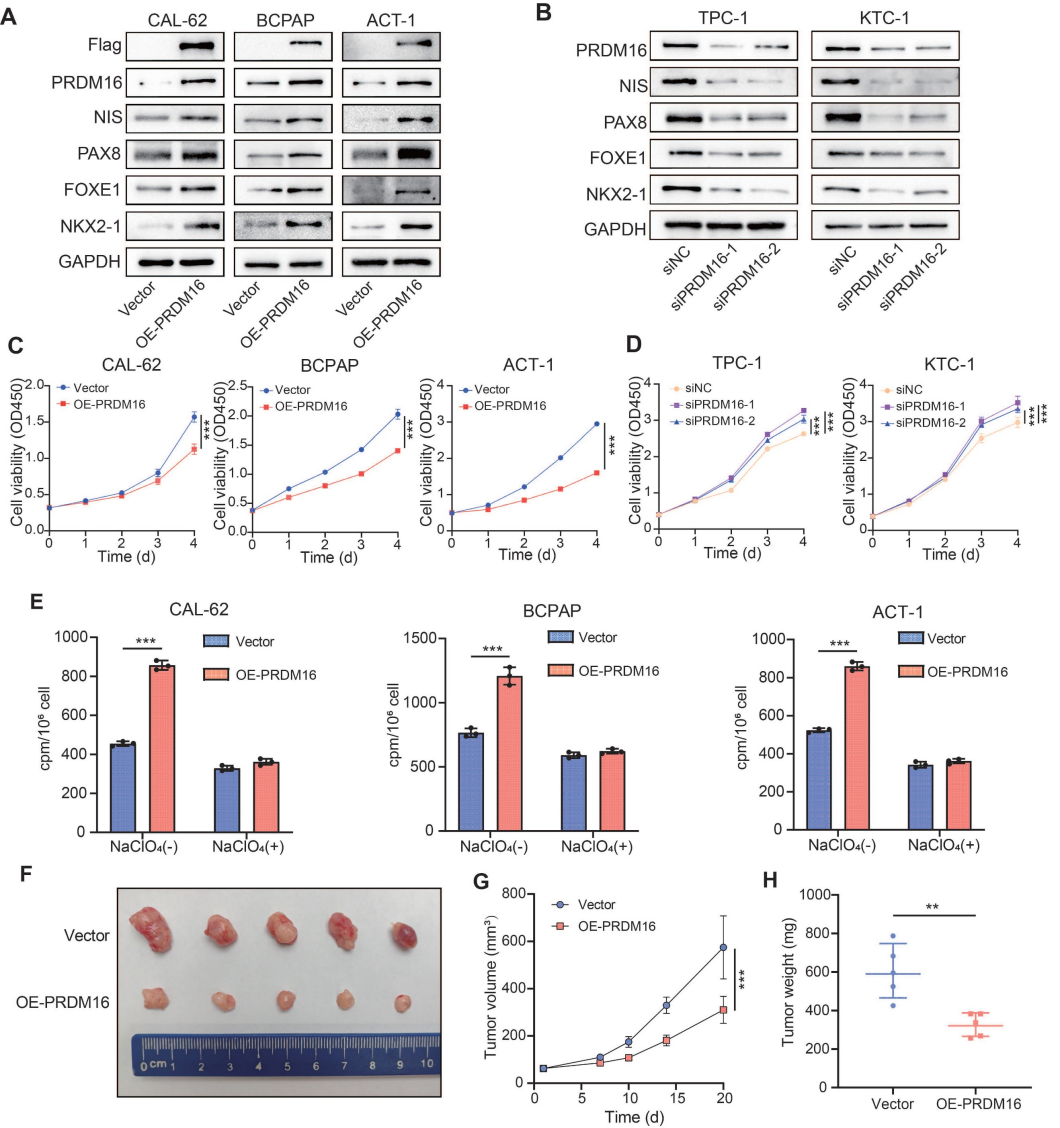
** PRDM16 facilitates the differentiation of thyroid cancer *in vitro* and *in vivo*.** (A, B) Western blotting analysis of thyroid differentiation marker expression following PRDM16 overexpression (A) or knockdown (B) in thyroid cancer cells. (C, D) CCK-8 assays assessing the proliferation of thyroid cancer cells with PRDM16 overexpression (C) or knockdown (D). (E) Radioactive iodine uptake assays determining the effect of PRDM16 overexpression on the iodine uptake capacity of thyroid cancer cells. (F) Image of subcutaneous xenografts derived from PRDM16-overexpressing or control CAL-62 cells (n = 5 per group). (G) Tumor growth curves of xenografts in PRDM16-overexpressing and control groups. (H) Comparison of tumor weights between PRDM16-overexpressing and control groups. All data represent mean ± SD. Statistical significance was determined by unpaired Student's t-test (C-E, G, and H).

To further investigate the regulatory role of PRDM16 in thyroid cancer differentiation, we overexpressed PRDM16 in three thyroid cancer cell lines (CAL-62, BCPAP, and ACT-1) with relatively low endogenous PRDM16 expression levels (Figure 2A). Additionally, we knocked down PRDM16 using siRNA in two thyroid cancer cell lines (TPC-1 and KTC-1) that exhibit relatively high endogenous PRDM16 expression levels (Figure 2B). The results demonstrated that PRDM16 overexpression promoted the expression of thyroid cancer differentiation markers (Figure 2A, Figure S2A), inhibited tumor cell proliferation, reduced colony-forming capacity (Figure 2C, Figure S2G), and enhanced radioactive iodine uptake (Figure 2E). Conversely, PRDM16 knockdown suppressed the expression of thyroid cancer differentiation markers (Figure 2B, Figure S2B), while promoting tumor cell proliferation and colony-forming capacity (Figure 2D, Figure S2H). PRDM16 has been shown to possess intrinsic histone methyltransferase activity that catalyzes monomethylation of histone H3 lysine 9. We therefore examined how PRDM16 knockdown or overexpression affects global H3K9me1 abundance. Our findings demonstrate that changes in PRDM16 expression levels directly modulate global H3K9me1 abundance in thyroid cancer cells (Figure S2C, D).

Given the critical role of tumor differentiation in regulating epithelial-mesenchymal transition (EMT), we measured EMT markers in both PRDM16-overexpressing and PRDM16-knockdown thyroid cancer cells. The results demonstrated that PRDM16 overexpression upregulated epithelial differentiation markers (E-cadherin) while downregulating mesenchymal differentiation markers (N-cadherin, Snail, and Slug) (Figure S2C), reducing thyroid cancer cell migration and invasion capabilities (Figure S2E, Figure S2I). Conversely, PRDM16 knockdown elevated mesenchymal differentiation marker expression, decreased epithelial differentiation markers (Figure S2D), and enhanced tumor cell migratory and invasive potential (Figure S2F, Figure S2J).

To investigate the ability of PRDM16 to facilitate the differentiation of thyroid cancer cells *in vivo*, we established tumor xenograft models by subcutaneously injecting CAL-62 cells into nude mice (Figure 2F). Compared to the control group, xenograft tumors derived from PRDM16-overexpressing CAL-62 cells exhibited significantly slower growth rates, along with markedly reduced tumor volume and weight (Figure 2G, H). We next performed IHC staining of the xenograft tumors to confirm PRDM16 overexpression *in vivo*. Compared with controls, PRDM16 overexpression resulted in decreased expression of the proliferation marker Ki67 and increased expression of differentiation markers (NIS, PAX8) (Figure S2K, L). In summary, PRDM16 promotes thyroid cancer cell differentiation and participates in regulating various biological behaviors of thyroid carcinoma.

### PRDM16-Mediated H3K9me1 Promotes TRIM58 Expression

**Figure 3:**
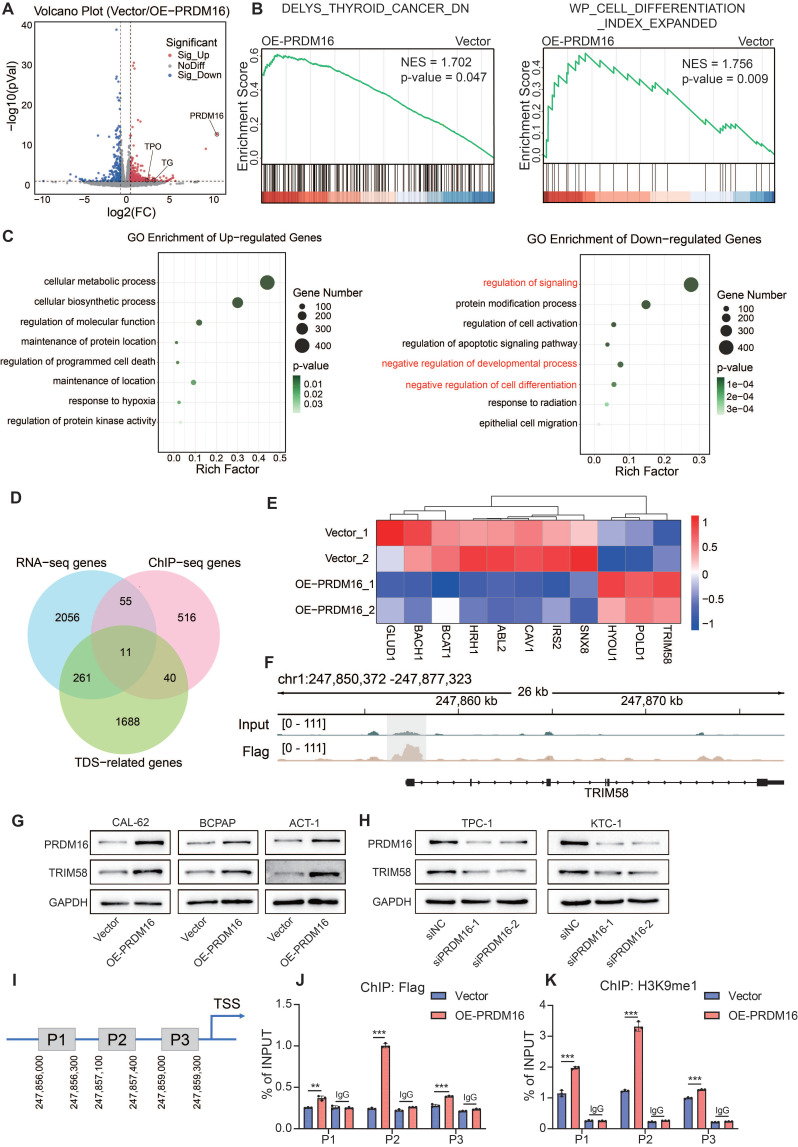
** TRIM58 was identified as a downstream molecule regulated by PRDM16.** (A) Volcano plot showing the differentially expressed genes identified by RNA-seq analysis in PRDM16-overexpressing CAL-62 cells. (B) GSEA indicating that the differentially expressed genes between PRDM16-overexpressing and control cells were significantly enriched in gene sets associated with thyroid cancer and thyroid dedifferentiation. (C) Representative GO term enrichment analyses of upregulated and downregulated genes following PRDM16 overexpression. (D) Venn diagram showing 11 overlapping genes identified among the RNA-seq-derived differentially expressed genes under PRDM16 overexpression, the ChIP-seq-identified PRDM16-binding genes, and the top 2000 genes most strongly correlated with the TDS. (E) Heatmap of RNA-seq data displaying the expression profiles of the 11 overlapping genes in PRDM16-overexpressing and control CAL-62 cells. (F) IGV analysis showing Flag enrichment near the TRIM58 promoter region in CAL-62 cells expressing PRDM16-3×Flag, compared with Input controls. (G, H) Western blot analysis showing changes in TRIM58 expression upon PRDM16 overexpression (G) or knockdown (H). (I) Schematic illustration of the ChIP-qPCR primer locations designed to target TRIM58 promoter regions. (J, K) ChIP-qPCR showing the enrichment levels of Flag (J) and H3K9me1 (K) in different regions of the TRIM58 promoter in PRDM16-3×Flag-overexpressing and control CAL-62 cells. All data represent mean ± SD. Statistical significance was determined by unpaired Student's t-test (J and K).

To further elucidate the intrinsic mechanism by which PRDM16 promotes differentiation in thyroid cancer, we performed RNA sequencing on both control cells and PRDM16-overexpressing cells to identify differentially expressed genes. PRDM16 overexpression upregulated 920 genes while downregulating 1461 genes (Figure 3A). Gene Set Enrichment Analysis (GSEA) revealed that PRDM16 overexpression was significantly associated with downregulated gene sets in thyroid cancer and showed a strong correlation with the cell differentiation index (Figure 3B). We performed Gene Ontology (GO) enrichment analysis on the differentially expressed genes. The results revealed that PRDM16 is involved in regulating multiple critical biological processes, including regulation of signaling, cell development, and cell differentiation, indicating its essential role in thyroid cancer differentiation (Figure 3C). To investigate the mechanism by which PRDM16 regulates gene expression, we performed ChIP sequencing (ChIP-seq) to identify direct target genes of PRDM16 in thyroid cancer (Figure S3A). We subsequently identified 11 key genes by intersecting differentially expressed genes, PRDM16 ChIP-seq targets, and the top 2000 genes most strongly correlated with TDS (Figure 3D). RNA-seq results demonstrated that TRIM58 was significantly upregulated in PRDM16-overexpressing cells among these 11 candidate genes, with clustering analysis further confirming its marked differential expression between the control and PRDM16-overexpressing groups (Figure 3E). Subsequent qPCR analysis of the 11 candidate genes verified that TRIM58 was significantly upregulated in PRDM16-overexpressing cells (Figure S3B). ChIP-seq analysis further revealed distinct peaks at the TRIM58 promoter region in the PRDM16-overexpressing group (Figure 3F). Correlation analysis revealed that the expression of TRIM58 was positively correlated with PRDM16 expression (Figure S3C). Moreover, TRIM58 expression was also positively correlated with the TDS in TCGA-THCA samples, indicating that higher TRIM58 levels are associated with a more differentiated tumor phenotype (Figure S3D). We speculate that PRDM16 may promote thyroid cancer differentiation by upregulating TRIM58 expression at the transcriptional level.

Then, we verified whether PRDM16 has a regulatory effect on TRIM58. The results showed that in thyroid cancer cells, overexpression of PRDM16 promoted TRIM58 expression (Figure 3G, Figure S3E), while knockdown of PRDM16 suppressed TRIM58 expression (Figure 3H, Figure S3F). We designed primers spanning the proximal promoter region of TRIM58 (Figure 3I). ChIP-qPCR results demonstrated that PRDM16 binds to the promoter region of TRIM58 and leads to increased enrichment of H3K9me1 at this specific region (Figure 3J, K). Considering that PRDM16 has been reported as a transcriptional regulator 34, we hypothesized that PRDM16 directly activates TRIM58 transcription. However, dual-luciferase reporter assays revealed that PRDM16 does not increase TRIM58 promoter activity (Figure S3G), thereby ruling out a direct transcriptional activation mechanism. In summary, we demonstrate that PRDM16 upregulates TRIM58 expression through epigenetic regulation, specifically by promoting H3K9me1 enrichment at its promoter.

### TRIM58 Facilitates Thyroid Cancer Differentiation

**Figure 4:**
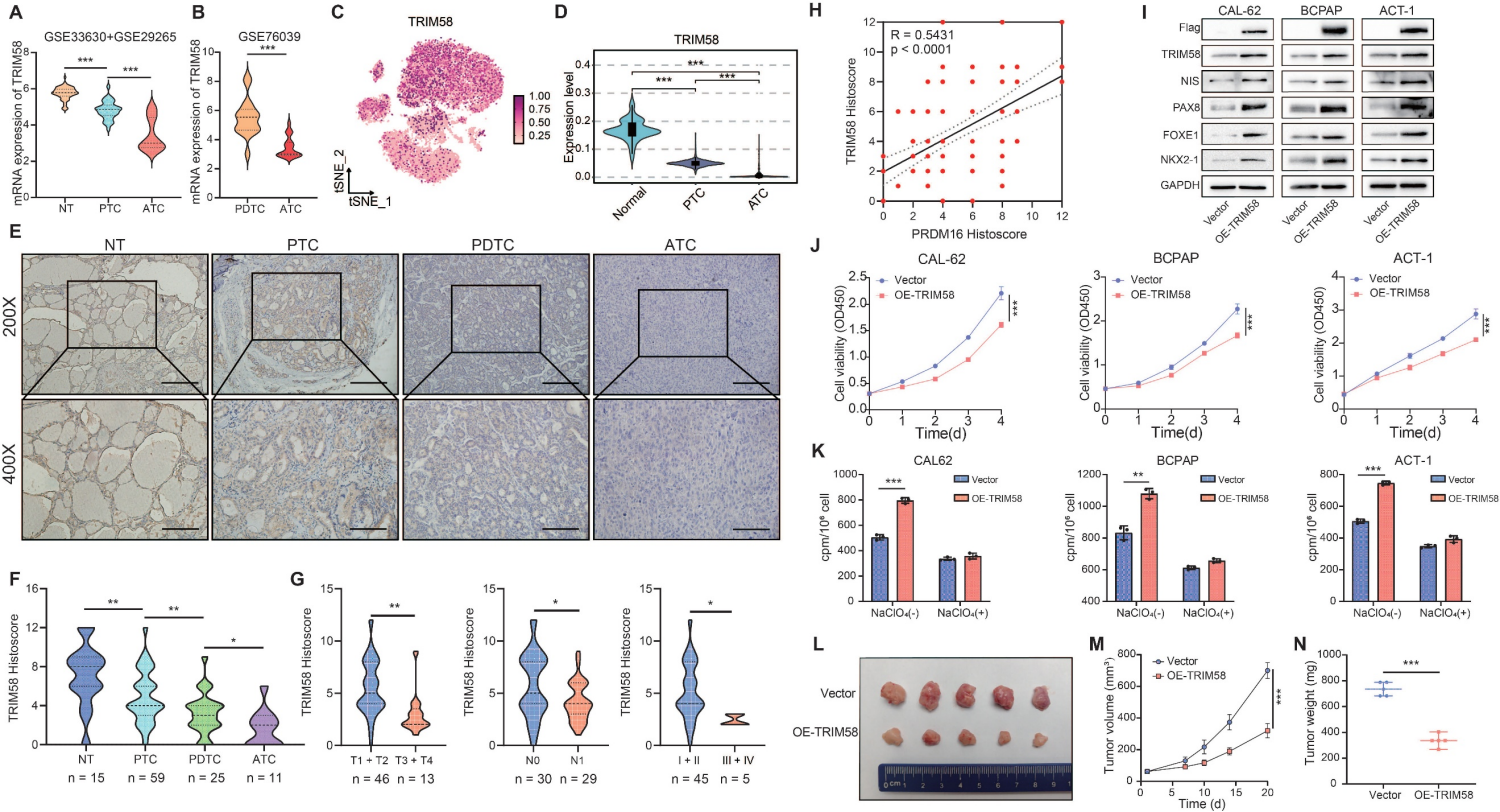
** TRIM58 facilitates the differentiation of thyroid cancer *in vitro* and *in vivo*.** (A, B) Expression analysis of TRIM58 in normal thyroid tissue and thyroid carcinoma samples from the GSE33630 + GSE29265 (A) and GSE76039 (B) datasets. (C) Uniform manifold approximation and projection (UMAP) plot displaying TRIM58 expression across thyroid carcinoma single-cell clusters. (D) Boxplot showing TRIM58 mRNA levels in NT, PTC, PDTC, and ATC tissues. (E) Representative IHC staining of TRIM58 in NT, PTC, PDTC, and ATC tissues. Scale bars = 200 μm. (F) Quantitative analysis of TRIM58 IHC histoscores across different thyroid cancer subtypes. (G) Correlation of TRIM58 immunohistochemical staining with tumor size, lymph node metastasis, and clinical stage in PTC patients. (H) Pearson's correlation analysis between PRDM16 and TRIM58 immunohistochemical histoscores. (I) Western blotting analysis of TRIM58 and thyroid differentiation markers following TRIM58 overexpression in CAL-62, BCPAP, and ACT-1 cells. (J) CCK-8 assay showing the effect of TRIM58 overexpression on the proliferation of CAL-62, BCPAP, and ACT-1 cells. (K) Radioactive iodine uptake assays evaluating the effect of TRIM58 overexpression on iodine uptake capacity in thyroid cancer cells. (L) Image of subcutaneous xenografts in nude mice derived from TRIM58-overexpressing or control CAL-62 cells (n = 5 for each group). (M) Growth curves of the subcutaneous xenografts in the TRIM58-overexpressing group and control group. (N) Analysis of the tumor weights of xenografts in the TRIM58-overexpressing group and control group. All data represent mean ± SD. Statistical significance was determined by unpaired Student's t-test (A, B, D, F, G, J-K, M, and N) or Pearson's correlation (H).

Given these observations, we next analyzed the expression of TRIM58 in thyroid cancer. Analysis of the TCGA database, CCLE database, and GEO datasets revealed that TRIM58 is expressed at lower levels in thyroid tumor cells (Figure S3H), and its expression further decreases as tumor differentiation declines (Figure 4A, B, Figure S3I). The expression level of TRIM58 is closely associated with the clinical characteristics of thyroid cancer patients (Figure S3J). Analysis of the single-cell dataset revealed that compared to normal thyroid follicular epithelial cells, the expression of TRIM58 was significantly decreased in thyroid cancer, with the lowest expression observed particularly in ATC (Figure 4C, D). Moreover, we examined the expression pattern of TRIM58 in normal tissues (n = 15), PTC tissues (n = 59), PDTC tissues (n = 25), and ATC tissues (n = 11) using IHC staining (Figure 4E). The results showed that TRIM58 expression decreases with the reduction in tumor differentiation (Figure 4F), and is closely associated with T stage, N stage, and clinical stage (Figure 4G). Additionally, paired analysis of IHC scoring revealed a strong positive correlation between TRIM58 and PRDM16 expression (Figure 4H).

To further explore how TRIM58 regulates thyroid cancer differentiation, we overexpressed TRIM58 in thyroid cancer cells. The results showed that overexpression of TRIM58 promoted the expression of thyroid differentiation markers (Figure 4I, Figure S4A). Additionally, TRIM58 overexpression suppressed the proliferation, colony formation, migration, and invasion of thyroid cancer cells (Figure 4J, Figure S4B-D), altered the expression of corresponding EMT markers (Figure S4E), and enhanced radioactive iodine uptake (Figure 4K).

Finally, to validate the ability of TRIM58 to promote differentiation of thyroid cancer cells *in vivo*, we established tumor xenograft models by subcutaneously injecting CAL-62 cells overexpressing TRIM58 into nude mice. Compared with the control group, the xenograft tumors derived from TRIM58-overexpressing thyroid cancer cells exhibited weaker proliferation capacity (Figure 4L), as evidenced by reduced tumor volume and weight (Figure 4M, N). The IHC staining of xenograft tissues confirmed TRIM58 overexpression *in vivo*, resulting in decreased proliferation markers and increased differentiation markers (Figure S4F, G).

### PRDM16 Promotes Thyroid Cancer Differentiation by Upregulating TRIM58 through Its Methyltransferase Activity

**Figure 5:**
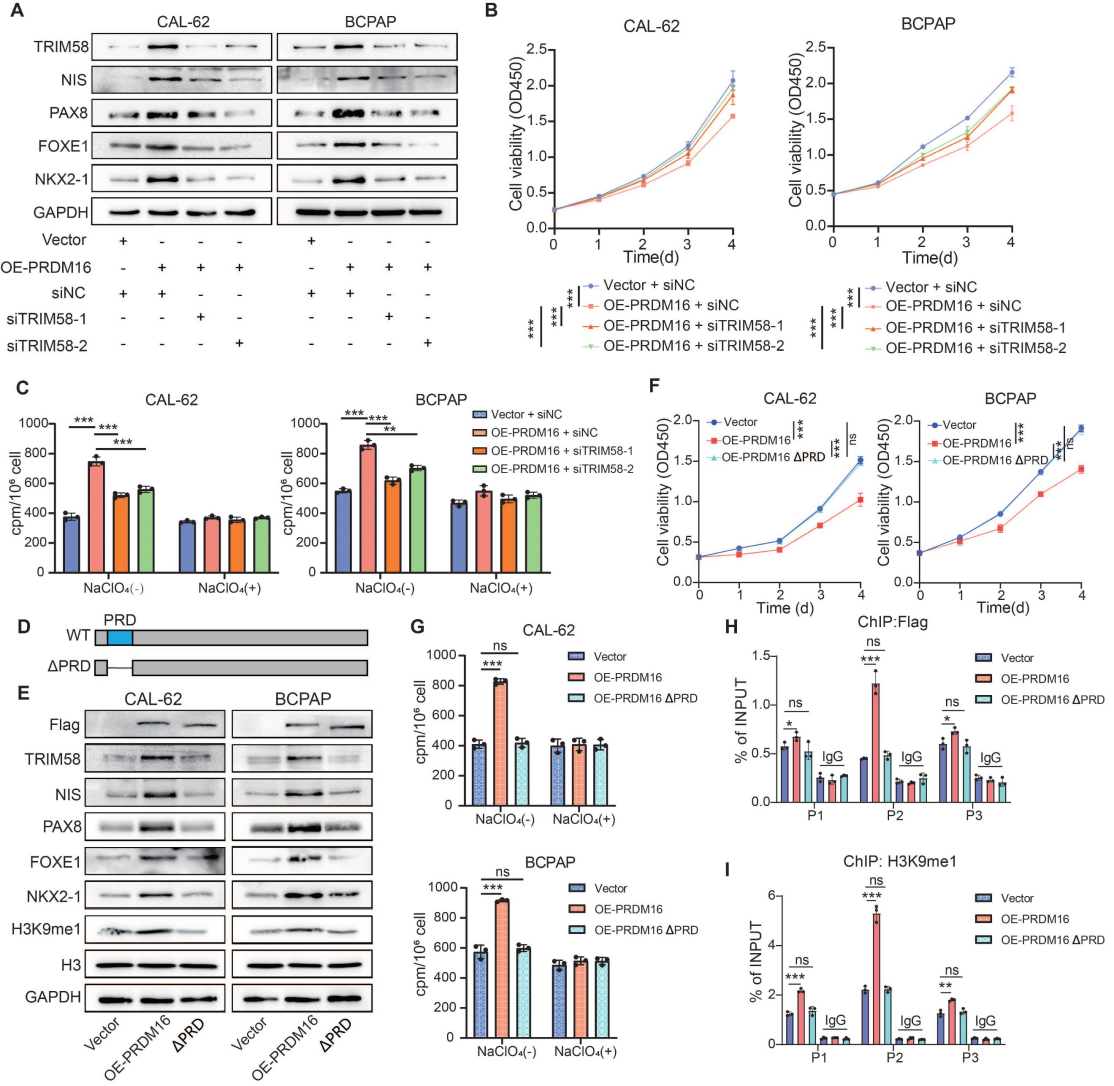
** PRDM16 promotes thyroid cancer differentiation by regulating TRIM58 through its methyltransferase activity.** (A) Western blotting analysis of thyroid differentiation marker expression levels in PRDM16-overexpressing cells with or without TRIM58 knockdown. (B) CCK-8 assays showing the effects of TRIM58 knockdown on cell proliferation in PRDM16-overexpressing thyroid cancer cells. (C) Radioactive iodine uptake assays determining the effect of TRIM58 knockdown on the iodine uptake capacity of PRDM16-overexpressing thyroid cancer cells. (D) Schematic diagram showing the location of the PR domain within the PRDM16 protein structure. (E) Western blotting analysis of thyroid differentiation marker and TRIM58 expression in thyroid cancer cells overexpressing wild-type PRDM16 or PR domain-deficient mutant PRDM16. (F) CCK-8 assay showing the effect of wild-type PRDM16 (WT) and PR domain-deficient PRDM16 overexpression on the proliferation of thyroid cancer cells. (G) Radioactive iodine uptake assay evaluating the effect of wild-type PRDM16 and PR domain-deficient PRDM16 overexpression on the iodine uptake capacity of thyroid cancer cells. (H, I) ChIP-qPCR analysis of Flag (H) and H3K9me1 (I) enrichment at different regions of the TRIM58 promoter in CAL-62 cells overexpressing wild-type PRDM16 or PR domain-deficient PRDM16. All data represent mean ± SD. Statistical significance was determined by unpaired Student's t-test (B, C, F-I).

To verify that PRDM16 promotes thyroid cancer differentiation through regulating TRIM58, we transfected TRIM58 siRNA into PRDM16-overexpressing thyroid cancer cells. The results showed that knockdown of TRIM58 could reverse the upregulation of thyroid differentiation markers, the upregulation of epithelial differentiation markers, and downregulation of mesenchymal differentiation markers induced by PRDM16 overexpression (Figure 5A, Figure S5A, B). Moreover, it concurrently reversed the suppression of proliferation, colony formation, migration, and invasion (Figure 5B, Figure S5C-E), as well as the enhanced radioiodine uptake capacity caused by PRDM16 overexpression (Figure 5C).

To further evaluate the role of PRDM16's methyltransferase activity in thyroid cancer differentiation, we overexpressed the methyltransferase-deficient mutant PRDM16 ΔPRD in thyroid cancer cells (Figure 5D). Compared to overexpression of wild-type PRDM16, overexpression of PRDM16 ΔPRD failed to promote the expression of TRIM58 and thyroid differentiation markers (Figure 5E, Figure S5F), and could not inhibit thyroid cancer cell proliferation, colony formation, migration, and invasion or enhance radioactive iodine uptake capacity (Figure 5F, G, Figure S5G, H). Furthermore, ChIP-qPCR results showed that, compared with wild-type PRDM16, PRDM16ΔPRD overexpression led to markedly reduced binding of PRDM16 to the TRIM58 promoter and decreased H3K9me1 enrichment at this locus (Figure 5H, I).

### TRIM58 Promotes Thyroid Cancer Differentiation by Ubiquitinating MVP

**Figure 6:**
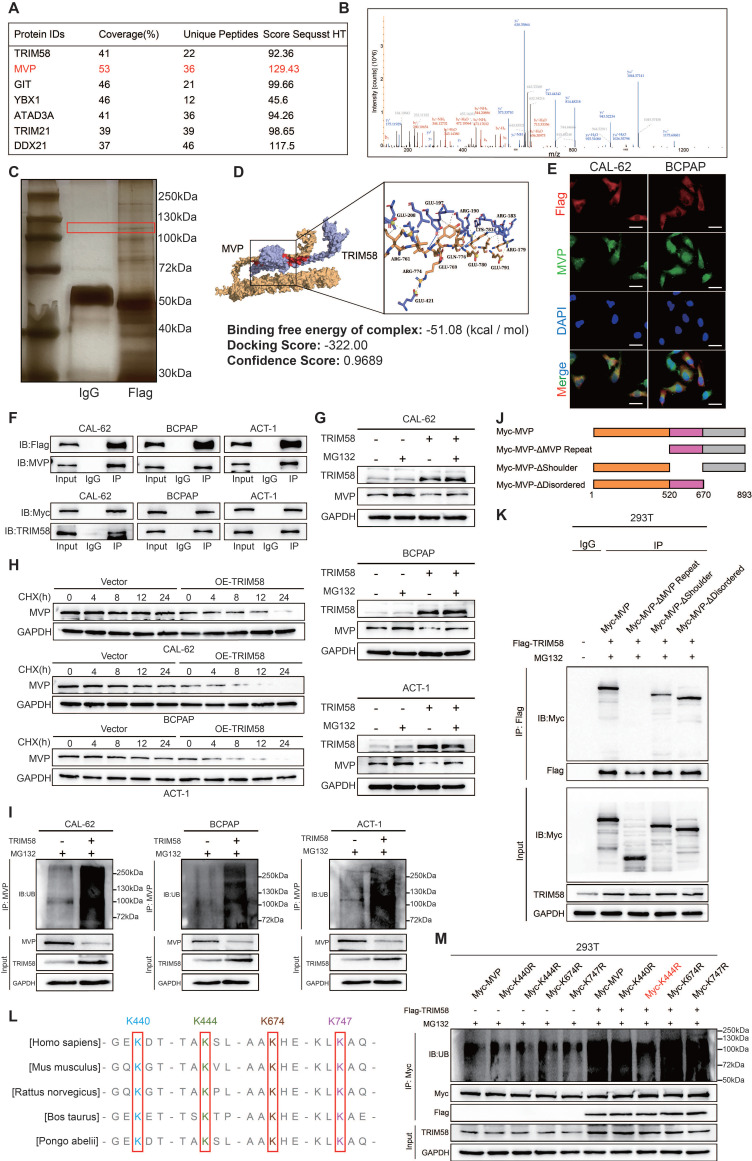
** TRIM58 mediates the ubiquitination and degradation of MVP protein.** (A) Immunoprecipitation combined with mass spectrometry (IP-MS) analysis of TRIM58-interacting proteins. (B) Peptide fragment mass spectrum of MVP protein. (C) Silver staining of proteins co-immunoprecipitated with Flag-tagged TRIM58. The band corresponding to MVP is indicated by a red box. (D) Molecular docking for predicting the interaction between TRIM58 and MVP. (E) Immunofluorescence staining showing colocalization of Flag-TRIM58 and MVP in thyroid cancer cells. Nuclei were counterstained with DAPI. Scale bars = 20 μm. (F) Co-IP assays confirming the interaction between TRIM58 and MVP. (G) Western blotting analysis of the effect of TRIM58 on MVP protein expression in thyroid cancer cells under the treatment of the proteasome inhibitor MG132. Cells were pretreated with 10 μM MG132 for 6 h before harvest. (H) Western blotting analysis of MVP protein expression in thyroid cancer cells treated with 10 μM cycloheximide (CHX) for varying time periods to assess the effect of TRIM58 on MVP protein stability. (I) Western blotting analysis validating the effect of TRIM58 on the ubiquitination level of MVP protein in thyroid cancer cells. Cells were pretreated with 10 μM MG132 for 6 h before harvest. (J) Schematic diagram of the full-length and truncated MVP structures. (K) Western blotting analysis of Co-IP results following co-transfection of Myc-tagged MVP or its truncated mutants with Flag-tagged TRIM58 into HEK293T cells. (L) Multiple sequence alignment of MVP protein sequences across diverse species; the predicted ubiquitination lysine residues are marked by red boxes. (M) Western blotting analysis assessing the ubiquitination of MVP in HEK293T cells co-transfected with Flag-TRIM58 and Myc-tagged MVP or its ubiquitination site mutants.

As a TRIM family member, TRIM58 possesses E3 ubiquitin ligase activity. To investigate its potential role in thyroid cancer differentiation, we performed Co-IP followed by liquid chromatography-tandem mass spectrometry (LC-MS/MS) to identify TRIM58-associated proteins. MVP was identified in the Flag-tagged TRIM58 immunoprecipitates (Figure 6A-C). MVP, a primary component of the ribonucleoprotein particles known as vaults, is recognized for its role in regulating a variety of cellular processes, including signal transduction, cell differentiation, and immune responses 35,36. Studies have reported that MVP is associated with various types of cancer and the development of chemotherapy resistance 37-40. Furthermore, MVP functions as a key regulator of the MAPK and PI3K/AKT signaling pathway in thyroid cancer. 41 Analysis of the GEO database revealed that MVP exhibits higher expression in poorly differentiated thyroid cancers, particularly in ATC (Figure S6A). Molecular docking predictions revealed a high binding probability between TRIM58 and MVP (Figure 6D). Moreover, immunofluorescence (IF) assays further demonstrated that TRIM58 and MVP colocalize in the cytoplasm (Figure 6E). To further characterize their subcellular distribution, nuclear and cytoplasmic fractionation assays were performed. Western blot analysis revealed that TRIM58 was predominantly localized in the cytoplasm, whereas MVP was detected in both the nucleus and cytoplasm. These results further support the cytoplasmic colocalization of TRIM58 and MVP (Figure S6B). Subsequently, we validated the interaction between TRIM58 and MVP using Co-IP assays. In thyroid tumor cells overexpressing Flag-TRIM58, MVP was co-immunoprecipitated with Flag-tagged TRIM58. Conversely, in cells overexpressing MVP, TRIM58 was co-immunoprecipitated with Myc-tagged MVP (Figure 6F), confirming a protein-protein interaction between TRIM58 and MVP.

We found that overexpression of TRIM58 in thyroid cancer cells led to a decrease in MVP protein levels but did not affect MVP transcriptional levels (Figure S6C-E). To further investigate whether TRIM58 regulates MVP through the proteasomal pathway, we first treated thyroid cancer cells from both the control and TRIM58-overexpressing groups with MG132. MG132 treatment markedly attenuated the TRIM58-induced reduction in MVP protein levels (Figure 6G). We next assessed the impact of TRIM58 overexpression on the stability of endogenous MVP. Following cycloheximide (CHX) treatment, TRIM58 overexpression significantly shortened the half-life of MVP compared with the control (Figure 6H). Finally, ubiquitination assays revealed that TRIM58 overexpression increased MVP ubiquitination, leading to decreased MVP protein levels (Figure 6I).

To map the TRIM58-MVP interaction domains, we constructed three Myc-tagged MVP truncation mutants and performed co-transfection with Flag-tagged TRIM58 (Figure 6J). Co-immunoprecipitation assays showed that TRIM58 specifically interacts with the ΔMVP Repeat fragment (Figure 6K). Having defined the MVP domain responsible for TRIM58 binding, we next sought to determine the specific ubiquitination sites on MVP targeted by TRIM58. PhosphoSitePlus analysis predicted four candidate lysine residues (K440, K444, K674, K747), which are highly conserved across species (Figure 6L). Site-directed mutagenesis demonstrated that substitution of K444 with arginine markedly reduced MVP ubiquitination (Figure 6M). Together, these results demonstrate that TRIM58 binds the ΔMVP Repeat domain of MVP and promotes its ubiquitin-mediated degradation primarily through K444, thereby facilitating thyroid cancer cell differentiation.

### PRDM16 Reinforces the Redifferentiation Effects of MAPK Inhibition

**Figure 7:**
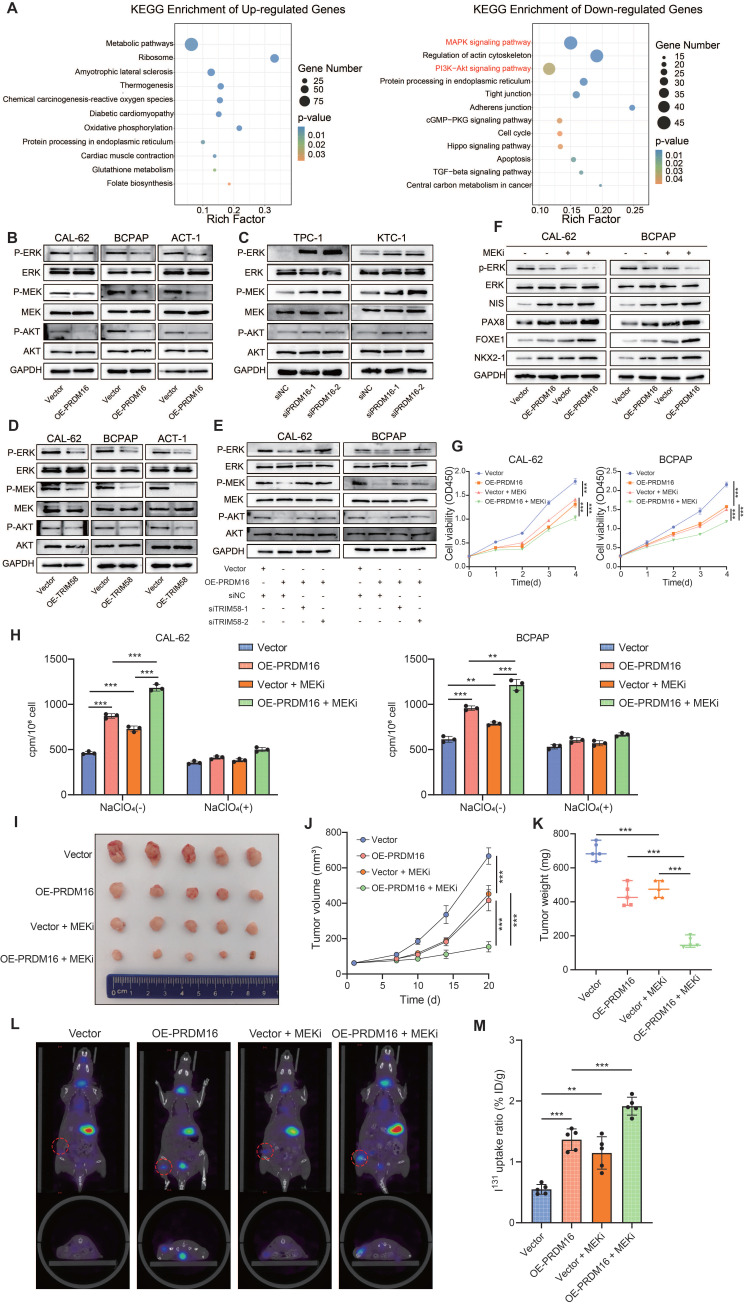
** PRDM16 reinforces the redifferentiation effects of MAPK inhibition.** (A) Representative KEGG term analysis of upregulated and downregulated genes was performed following PRDM16 overexpression. (B, C) Western blotting analysis of the effects of PRDM16 overexpression (B) or knockdown (C) on MAPK and PI3K/AKT pathway activity in thyroid cancer cells. (D) Western blotting analysis of the effects of TRIM58 overexpression on MAPK and PI3K/AKT pathway activity in thyroid cancer cells. (E) Western blotting analysis of MAPK and PI3K/AKT pathway activity following TRIM58 knockdown in PRDM16-overexpressing thyroid cancer cells. (F) Western blotting analysis of p-ERK, total ERK, and thyroid differentiation marker expression in PRDM16-overexpressing thyroid cancer cells with or without 1 μM selumetinib treatment for 24 h. (G) CCK-8 assays showing the effects of 1 μM selumetinib treatment on cell proliferation in PRDM16-overexpressing thyroid cancer cells. (H) Radioactive iodine uptake assays showing the effects of 1 μM selumetinib treatment for 24 h on iodine uptake in thyroid cancer cells with or without PRDM16 overexpression. (I) Subcutaneous nude mouse xenografts formed by PRDM16-overexpressing or control CAL-62 cells were photographed after treatment with or without selumetinib. When the average tumor volume reached 50 mm^3^, selumetinib was intragastrically administered at a dose of 10 mg/kg once daily. n = 5 animals per group. Subcutaneous xenografts were dissected on Day 20 and analyzed. (J) Growth curves of the subcutaneous xenografts in each group. (K) Tumor weights of each group. (L) Representative *in vivo* SPECT/CT images showing ¹³¹I distribution in tumor xenografts (red dashed circles) and thyroid glands after radioiodine administration. (M) Normalized radioiodine uptake in xenograft tumors. Uptake was expressed as %ID/g. All data represent mean ± SD. Statistical significance was determined by unpaired Student's t-test (G, H, J, K, and M).

Considering that MVP regulates both MAPK and PI3K/AKT signaling pathways 41, we sought to determine whether PRDM16 promotes thyroid cancer cell differentiation by suppressing the activity of these two pathways. To this end, we re-analyzed the RNA-seq data from PRDM16-overexpressing cells. KEGG enrichment analysis of differentially expressed genes revealed that the downregulated genes were significantly enriched in both the MAPK and PI3K/AKT signaling pathways (Figure 7A). To investigate whether PRDM16 suppresses the MAPK and PI3K/AKT pathways through TRIM58-mediated ubiquitination and degradation of MVP, we first assessed the activity of both pathways following PRDM16 overexpression or knockdown. PRDM16 overexpression markedly suppressed both MAPK and PI3K/AKT signaling, whereas PRDM16 knockdown enhanced their activity (Figure 7B, C). Consistently, TRIM58 overexpression produced a similar inhibitory effect on both pathways (Figure 7D). Moreover, TRIM58 knockdown in PRDM16-overexpressing cells reversed the inhibitory effect of PRDM16 on MAPK and PI3K/AKT signaling (Figure 7E). To further verify the effect of MVP on thyroid cancer cell differentiation and the activation of these two signaling pathways in the PRDM16-TRIM58 regulatory axis, rescue experiments were conducted in PRDM16-overexpressing cells by restoring MVP expression. The results showed that MVP restoration reversed the upregulation of differentiation markers induced by PRDM16 overexpression and significantly activated the two signaling pathways (Figure S7A). Additionally, MVP restoration also reversed the inhibitory effects of PRDM16 overexpression on cell proliferation, migration, and invasion (Figure S7B, C).

Given that MAPK inhibitors can promote the redifferentiation capacity of thyroid cancer but often exhibit low therapeutic sensitivity, we further investigated whether PRDM16 influences the sensitivity of thyroid cancer to MAPK inhibitor-based redifferentiation therapy. Our study revealed that PRDM16 overexpression potently enhanced the therapeutic redifferentiation effect of the MAPK inhibitor selumetinib, which suppressed the MAPK pathway and promoted the expression of thyroid differentiation markers (Figure 7F). Moreover, it significantly inhibited the proliferation, colony formation (Figure 7G, Figure S7D), migration, and invasion capabilities of thyroid cancer cells (Figure S7E). Furthermore, PRDM16 overexpression potently amplified the MAPK inhibitor-induced radioactive iodine uptake capacity (Figure 7H).

Next, we evaluated the impact of PRDM16 on the redifferentiation function of MAPK inhibitors *in vivo*. We established tumor xenograft models by subcutaneously injecting CAL-62 control cells and PRDM16-overexpressing cells into nude mice. When the average tumor volume reached 50 mm^3^, the mice were administered 10 mg/kg selumetinib once daily via intragastric gavage. The subcutaneous xenografts were dissected on Day 20. The results demonstrated that PRDM16 overexpression significantly enhanced the antiproliferative efficacy of MAPK inhibitors (Figure 7I), as evidenced by reduced tumor volume and weight (Figure 7J, K). IHC staining of the xenograft tumors revealed that PRDM16 overexpression promoted a reduction in tumor proliferation markers and an increase in thyroid differentiation markers induced by MAPK inhibitors *in vivo* (Figure S7F, G). Finally, we evaluated the effect of PRDM16 on the MAPK inhibitor-induced radioiodine uptake ability *in vivo*. A tumor xenograft model was established by subcutaneously injecting CAL-62 control cells and PRDM16-overexpressing cells into nude mice. Following selumetinib administration, *in vivo* SPECT/CT imaging and subsequent γ-counting demonstrated that PRDM16 overexpression significantly enhanced selumetinib-induced iodine uptake capacity in tumors, as evidenced by a significant increase in normalized tumor radioiodine uptake compared with the control group (Figure 7L, M). In summary, PRDM16 enhances the therapeutic efficacy of MAPK inhibitor redifferentiation therapy in thyroid cancer, suggesting its potential as a novel therapeutic target for patients with advanced thyroid cancer.

## Discussion

Thyroid cancer represents a paradigmatic model for studying lineage plasticity in epithelial malignancies, where the progressive loss of differentiation is closely linked to treatment resistance and poor prognosis. Although the genetic landscape of thyroid cancer has been well defined, encompassing canonical drivers such as BRAF^V600E, RAS, and TERT promoter mutations 42-45, the epigenetic and post-translational regulatory frameworks that maintain or disrupt thyroid cell identity remain poorly understood. In this study, we identified PRDM16 as a differentiation-associated histone methylation regulator that preserves thyroid lineage identity through a TRIM58-MVP/MAPK and PI3K/AKT signaling axis, providing a new conceptual model that integrates chromatin modification, ubiquitination, and signaling regulation in thyroid tumor dedifferentiation.

Epigenetic dysregulation has emerged as a critical driver of tumor dedifferentiation and therapy resistance across multiple cancer types. PRDM16, a PR domain-containing histone methyltransferase, modulates chromatin states through site-specific histone methylation to regulate cellular differentiation. Although previous studies have primarily characterized PRDM16 as a transcriptional regulator in processes such as brown adipogenesis and hematopoiesis 46,47, subsequent work has also demonstrated its role in shaping chromatin architecture and enhancer activity to establish lineage identity 48,49. In contrast, our findings revealed that, in thyroid cancer, PRDM16 exerts its function predominantly through its intrinsic histone methyltransferase activity, highlighting a distinct epigenetic mechanism underlying its regulatory role. PRDM16 expression is progressively reduced during thyroid cancer dedifferentiation and correlates positively with thyroid differentiation scores, suggesting that PRDM16 functions as a lineage fidelity factor that preserves the differentiated state of thyroid cells. Mechanistically, PRDM16 catalyzes H3K9me1 within the TRIM58 promoter, facilitating an active chromatin state and enhancing its transcription. Although H3K9me1 has traditionally been regarded as a repressive histone mark associated with transcriptional silencing 29,50,51, emerging evidence indicates that it can also function as a permissive or even activating modification depending on the genomic context, chromatin environment, and interacting cofactors 52-56. In this light, PRDM16-mediated H3K9me1 at the TRIM58 promoter likely represents such a context-dependent activating configuration, promoting chromatin accessibility and transcriptional activation. This observation underscores the nuanced regulatory roles of H3K9me1 in gene expression and highlights the epigenetic function of PRDM16 in maintaining thyroid cell differentiation.

The tripartite motif (TRIM) proteins function as important regulators in innate immunity, tumorigenesis, cell differentiation, and ontogenetic development 57. TRIM proteins, most of which possess E3 ubiquitin ligase activity, may regulate numerous oncogenes and tumor suppressors through ubiquitination and protein degradation, thereby influencing cancer progression 58. Previous studies have demonstrated that TRIM58 functions as a tumor suppressor in the majority of cancers, inhibiting tumor proliferation and invasion 59,60, and promotes the differentiation of breast cancer stem cells through ubiquitination 61. In thyroid cancer, however, its biological role has not been elucidated. Our study demonstrated that PRDM16 induces TRIM58 expression through H3K9me1-mediated epigenetic activation, establishing TRIM58 as a key effector of PRDM16-dependent differentiation. We showed that TRIM58 suppresses MAPK and PI3K/AKT pathway activity by promoting the ubiquitination and degradation of MVP, a scaffold known to facilitate sustained MAPK and PI3K/AKT signaling 41,62. TRIM58-mediated degradation of MVP results in attenuation of MAPK and PI3K/AKT signaling, thereby resulting in the restoration of thyroid-specific gene expression including NIS, PAX8, NKX2-1 and FOXE1, and reactivation of radioiodine uptake. This finding establishes a direct molecular bridge between PRDM16-dependent chromatin remodeling and post-translational control of oncogenic signaling, providing mechanistic clarity to how differentiation can be reinstated in otherwise refractory thyroid cancer cells.

Redifferentiation therapy aims to restore RAI sensitivity in refractory thyroid cancers by reactivating thyroid-specific gene expression 12,63. Our findings expand this model by revealing that PRDM16-driven histone methylation creates a chromatin-permissive environment that sustains redifferentiation. In this context, PRDM16 functions synergistically with MAPK inhibition, as its reactivation enhances the efficacy of MAPK inhibitors. Thus, the PRDM16-TRIM58-MVP axis represents an integrated mechanism that couples epigenetic stability with signaling plasticity, providing a more comprehensive framework for understanding redifferentiation therapy.

## Conclusions

In summary, our study demonstrates the crucial role of the PRDM16-TRIM58-MVP signaling axis in thyroid carcinoma differentiation. Each component of this axis may serve as a potential biomarker for thyroid cancer, and targeted intervention on these genes could potentially induce the redifferentiation of thyroid carcinoma. Furthermore, this research provides an important strategic framework for differentiation therapy in thyroid cancer treatment.

## Supplementary Material

Supplementary methods, figures and tables.

## Data Availability

All data generated or analyzed during this study are included in this published article and its supplementary information files. The datasets used and analysed during the current study are available from the corresponding author on reasonable request.

## References

[1] Chen DW, Lang BHH, McLeod DSA, Newbold K, Haymart MR (2023). Thyroid cancer. Lancet.

[2] Boucai L, Zafereo M, Cabanillas ME (2024). Thyroid Cancer: A Review. JAMA.

[3] Liu Y, Wang J, Hu X, Pan Z, Xu T, Xu J (2023). Radioiodine therapy in advanced differentiated thyroid cancer: Resistance and overcoming strategy. Drug Resist Updat.

[4] Lee DY, Won J-K, Lee S-H, Park DJ, Jung KC, Sung M-W (2016). Changes of Clinicopathologic Characteristics and Survival Outcomes of Anaplastic and Poorly Differentiated Thyroid Carcinoma. Thyroid.

[5] Ibrahimpasic T, Ghossein R, Carlson DL, Nixon I, Palmer FL, Shaha AR (2014). Outcomes in patients with poorly differentiated thyroid carcinoma. J Clin Endocrinol Metab.

[6] Liang J-A, Chen C-P, Huang S-J, Ho T-Y, Hsiang C-Y, Ding H-J (2005). A novel loss-of-function deletion in sodium/iodide symporter gene in follicular thyroid adenoma. Cancer Lett.

[7] Hou P, Bojdani E, Xing M (2010). Induction of Thyroid Gene Expression and Radioiodine Uptake in Thyroid Cancer Cells by Targeting Major Signaling Pathways. J Clin Endocrinol Metab.

[8] Medyouf H (2017). The microenvironment in human myeloid malignancies: emerging concepts and therapeutic implications. Blood.

[9] Dos Santos GA, Kats L, Pandolfi PP (2013). Synergy against PML-RARa: targeting transcription, proteolysis, differentiation, and self-renewal in acute promyelocytic leukemia. J Exp Med.

[10] Yan M, Zhang Y, He B, Xiang J, Wang Z, Zheng F (2014). IKKα restoration via EZH2 suppression induces nasopharyngeal carcinoma differentiation. Nat Commun.

[11] Matthay KK, Villablanca JG, Seeger RC, Stram DO, Harris RE, Ramsay NK (1999). Treatment of high-risk neuroblastoma with intensive chemotherapy, radiotherapy, autologous bone marrow transplantation, and 13-cis-retinoic acid. Children's Cancer Group. N Engl J Med.

[12] Oh JM, Ahn B-C (2021). Molecular mechanisms of radioactive iodine refractoriness in differentiated thyroid cancer: Impaired sodium iodide symporter (NIS) expression owing to altered signaling pathway activity and intracellular localization of NIS. Theranostics.

[13] Knauf JA, Ma X, Smith EP, Zhang L, Mitsutake N, Liao X-H (2005). Targeted expression of BRAFV600E in thyroid cells of transgenic mice results in papillary thyroid cancers that undergo dedifferentiation. Cancer Res.

[14] Zhang L, Xu S, Cheng X, Wu J, Wang X, Wu L (2021). Curcumin enhances the membrane trafficking of the sodium iodide symporter and augments radioiodine uptake in dedifferentiated thyroid cancer cells via suppression of the PI3K-AKT signaling pathway. Food Funct.

[15] He Y, Tang Z, Xu M, Huang T (2025). Dedifferentiation and Redifferentiation of Follicular-Cell-Derived Thyroid Carcinoma: Mechanisms and Therapeutic Implications. Biomedicines.

[16] Toro-Tobon D, Morris JC, Hilger C, Peskey C, Durski JM, Ryder M (2024). Clinical Outcomes of Radioactive Iodine Redifferentiation Therapy in Previously Iodine Refractory Differentiated Thyroid Cancers. Thyroid.

[17] Balakirouchenane D, Seban R, Groussin L, Puszkiel A, Cottereau AS, Clerc J (2023). Pharmacokinetics/Pharmacodynamics of Dabrafenib and Trametinib for Redifferentiation and Treatment of Radioactive Iodine-Resistant Mutated Advanced Differentiated Thyroid Cancer. Thyroid.

[18] Fenton MS, Marion KM, Salem AK, Hogen R, Naeim F, Hershman JM (2010). Sunitinib inhibits MEK/ERK and SAPK/JNK pathways and increases sodium/iodide symporter expression in papillary thyroid cancer. Thyroid.

[19] Ho AL, Grewal RK, Leboeuf R, Sherman EJ, Pfister DG, Deandreis D (2013). Selumetinib-enhanced radioiodine uptake in advanced thyroid cancer. N Engl J Med.

[20] Dunn LA, Sherman EJ, Baxi SS, Tchekmedyian V, Grewal RK, Larson SM (2019). Vemurafenib Redifferentiation of *BRAF* Mutant, RAI-Refractory Thyroid Cancers. The Journal of Clinical Endocrinology & Metabolism.

[21] Sun L, Zhang H, Gao P (2022). Metabolic reprogramming and epigenetic modifications on the path to cancer. Protein Cell.

[22] Audia JE, Campbell RM (2016). Histone Modifications and Cancer. Cold Spring Harb Perspect Biol.

[23] Hyun K, Jeon J, Park K, Kim J (2017). Writing, erasing and reading histone lysine methylations. Exp Mol Med.

[24] Noerenberg D, Damm F (2024). Beyond the code: the role of histone methylation in cancer resistance and therapy. Signal Transduct Target Ther.

[25] Tzatsos A, Paskaleva P, Ferrari F, Deshpande V, Stoykova S, Contino G (2013). KDM2B promotes pancreatic cancer via Polycomb-dependent and -independent transcriptional programs. J Clin Invest.

[26] Wong SHK, Goode DL, Iwasaki M, Wei MC, Kuo H-P, Zhu L (2015). The H3K4-Methyl Epigenome Regulates Leukemia Stem Cell Oncogenic Potential. Cancer Cell.

[27] Zhang W, Ruan X, Huang Y, Zhang W, Xu G, Zhao J (2024). SETMAR Facilitates the Differentiation of Thyroid Cancer by Regulating SMARCA2-Mediated Chromatin Remodeling. Adv Sci (Weinh).

[28] Hohenauer T, Moore AW (2012). The Prdm family: expanding roles in stem cells and development. Development.

[29] Pinheiro I, Margueron R, Shukeir N, Eisold M, Fritzsch C, Richter FM (2012). Prdm3 and Prdm16 are H3K9me1 methyltransferases required for mammalian heterochromatin integrity. Cell.

[30] Hurwitz E, Parajuli P, Ozkan S, Prunier C, Nguyen TL, Campbell D (2023). Antagonism between Prdm16 and Smad4 specifies the trajectory and progression of pancreatic cancer. J Cell Biol.

[31] Fei L-R, Huang W-J, Wang Y, Lei L, Li Z-H, Zheng Y-W (2019). PRDM16 functions as a suppressor of lung adenocarcinoma metastasis. J Exp Clin Cancer Res.

[32] Kundu A, Nam H, Shelar S, Chandrashekar DS, Brinkley G, Karki S (2020). PRDM16 suppresses HIF-targeted gene expression in kidney cancer. J Exp Med.

[33] van Dijk D, Sharma R, Nainys J, Yim K, Kathail P, Carr AJ (2018). Recovering Gene Interactions from Single-Cell Data Using Data Diffusion. Cell.

[34] Wu T, Liang Z, Zhang Z, Liu C, Zhang L, Gu Y (2022). PRDM16 Is a Compact Myocardium-Enriched Transcription Factor Required to Maintain Compact Myocardial Cardiomyocyte Identity in Left Ventricle. Circulation.

[35] Yuan L, Zhao N, Wang J, Liu Y, Meng L, Guo S (2021). Major vault protein (MVP) negatively regulates osteoclastogenesis via calcineurin-NFATc1 pathway inhibition. Theranostics.

[36] Berger W, Steiner E, Grusch M, Elbling L, Micksche M (2009). Vaults and the major vault protein: novel roles in signal pathway regulation and immunity. Cell Mol Life Sci.

[37] Teng Y, Ren Y, Hu X, Mu J, Samykutty A, Zhuang X (2017). MVP-mediated exosomal sorting of miR-193a promotes colon cancer progression. Nat Commun.

[38] Xiao Y-S, Zeng D, Liang Y-K, Wu Y, Li M-F, Qi Y-Z (2019). Major vault protein is a direct target of Notch1 signaling and contributes to chemoresistance in triple-negative breast cancer cells. Cancer Lett.

[39] Yu H, Li M, He R, Fang P, Wang Q, Yi Y (2020). Major Vault Protein Promotes Hepatocellular Carcinoma Through Targeting Interferon Regulatory Factor 2 and Decreasing p53 Activity. Hepatology.

[40] Shen W, Qiu Y, Li J, Wu C, Liu Z, Zhang X (2019). IL-25 promotes cisplatin resistance of lung cancer cells by activating NF-κB signaling pathway to increase of major vault protein. Cancer Med.

[41] Dong X, Akuetteh PDP, Song J, Ni C, Jin C, Li H (2021). Major Vault Protein (MVP) Associated With BRAF V600E Mutation Is an Immune Microenvironment-Related Biomarker Promoting the Progression of Papillary Thyroid Cancer via MAPK/ERK and PI3K/AKT Pathways. Front Cell Dev Biol.

[42] Landa I, Ibrahimpasic T, Boucai L, Sinha R, Knauf JA, Shah RH (2016). Genomic and transcriptomic hallmarks of poorly differentiated and anaplastic thyroid cancers. J Clin Invest.

[43] Nikiforov YE, Nikiforova MN (2011). Molecular genetics and diagnosis of thyroid cancer. Nat Rev Endocrinol.

[44] Cancer Genome Atlas Research Network (2014). Integrated genomic characterization of papillary thyroid carcinoma. Cell.

[45] Yu P, Qu N, Zhu R, Hu J, Han P, Wu J (2023). TERT accelerates BRAF mutant-induced thyroid cancer dedifferentiation and progression by regulating ribosome biogenesis. Sci Adv.

[46] Seale P, Bjork B, Yang W, Kajimura S, Chin S, Kuang S (2008). PRDM16 controls a brown fat/skeletal muscle switch. Nature.

[47] Aguilo F, Avagyan S, Labar A, Sevilla A, Lee D-F, Kumar P (2011). Prdm16 is a physiologic regulator of hematopoietic stem cells. Blood.

[48] Harms MJ, Lim H-W, Ho Y, Shapira SN, Ishibashi J, Rajakumari S (2015). PRDM16 binds MED1 and controls chromatin architecture to determine a brown fat transcriptional program. Genes Dev.

[49] He H, Bell SM, Davis AK, Zhao S, Sridharan A, Na C-L (2024). PRDM3/16 regulate chromatin accessibility required for NKX2-1 mediated alveolar epithelial differentiation and function. Nat Commun.

[50] Methot SP, Padeken J, Brancati G, Zeller P, Delaney CE, Gaidatzis D (2021). H3K9me selectively blocks transcription factor activity and ensures differentiated tissue integrity. Nat Cell Biol.

[51] Biferali B, Bianconi V, Perez DF, Kronawitter SP, Marullo F, Maggio R (2021). Prdm16-mediated H3K9 methylation controls fibro-adipogenic progenitors identity during skeletal muscle repair. Sci Adv.

[52] Zhang Y, Xue W, Zhang W, Yuan Y, Zhu X, Wang Q (2020). Histone methyltransferase G9a protects against acute liver injury through GSTP1. Cell Death Differ.

[53] Lu T, Yang J, Cai Y, Ding M, Yu Z, Fang X (2025). NCAPD3 promotes diffuse large B-cell lymphoma progression through modulating SIRT1 expression in an H3K9 monomethylation-dependent manner. J Adv Res.

[54] Barski A, Cuddapah S, Cui K, Roh T-Y, Schones DE, Wang Z (2007). High-resolution profiling of histone methylations in the human genome. Cell.

[55] Zhao Z, Shilatifard A (2019). Epigenetic modifications of histones in cancer. Genome Biol.

[56] Zhou H, Du Y, Wei X, Song C, Song J, Xu N (2022). DDX56 transcriptionally activates MIST1 to facilitate tumorigenesis of HCC through PTEN-AKT signaling. Theranostics. Ivyspring International Publisher.

[57] Gao Y, Pan T, Xu G, Li S, Guo J, Zhang Y (2022). Pan-cancer illumination of TRIM gene family reveals immunology regulation and potential therapeutic implications. Hum Genomics.

[58] Wang D, Ma L, Wang B, Liu J, Wei W (2017). E3 ubiquitin ligases in cancer and implications for therapies. Cancer Metastasis Rev.

[59] Liu X, Long Z, Cai H, Yu S, Wu J (2020). TRIM58 suppresses the tumor growth in gastric cancer by inactivation of β-catenin signaling via ubiquitination. Cancer Biol Ther.

[60] Liu M, Zhang X, Cai J, Li Y, Luo Q, Wu H (2018). Downregulation of TRIM58 expression is associated with a poor patient outcome and enhances colorectal cancer cell invasion. Oncol Rep.

[61] Li X, Jiang J, Wu Q, You T, Yang F (2024). TRIM58 downregulation maintains stemness via MYH9-GRK3-YAP axis activation in triple-negative breast cancer stem cells. Cancer Gene Ther.

[62] Liu Z, Zhang W, Phillips JB, Arora R, McClellan S, Li J (2019). Immunoregulatory protein B7-H3 regulates cancer stem cell enrichment and drug resistance through MVP-mediated MEK activation. Oncogene.

[63] Liu J, Liu Y, Lin Y, Liang J (2019). Radioactive Iodine-Refractory Differentiated Thyroid Cancer and Redifferentiation Therapy. Endocrinol Metab (Seoul).

