# Supplementary material for: PRDM16 Drives Thyroid Cancer Differentiation via a TRIM58-MVP Axis to Suppress MAPK and PI3K/AKT Signaling
Source: Int J Biol Sci. 2026 Apr 23;22(9):4747–64. doi: 10.7150/ijbs.131151 (PMC13182561; doi:10.7150/ijbs.131151)
Supplement: Supplementary file 1 — Supplementary methods, figures and tables. [file ijbsv22p4747s1.pdf]

**PRDM16 Drives Thyroid Cancer Differentiation via a TRIM58–MVP Axis to Suppress MAPK and PI3K/AKT Signaling**

Jialong Yu[1#], Wei Luo[1#], Zijie Niu[1#], Miao Zhang[1#], Yunqi Gao[2], Mengran Tian[2, 3], Zhongyu Wang[1], Xing Wan[1], Han Gui[4], Qian Su[5], Wenhao Chen[1], Linfei Hu[1], Xiukun Hou[1], Qiman Dong[1*], Xianhui Ruan[1*], Xiangqian Zheng[1*]

1 Department of Thyroid and Neck Tumor, Tianjin Medical University Cancer Institute and Hospital, Tianjin's Clinical Research Center for Cancer, Tianjin, China.

2 School of Medicine, Nankai University, Tianjin, China.

3 Department of Thyroid and Breast Surgery, Tianjin Union Medical Center, The First Affiliated Hospital of Nankai University, Tianjin, China.

4 State Key Laboratory of Advanced Medical Materials and Devices, Tianjin Key Laboratory of Radiation Medicine and Molecular Nuclear Medicine, Key Laboratory of Radiopharmacokinetics for Innovative Drugs, Tianjin Institutes of Health Science, Institute of Radiation Medicine, Chinese Academy of Medical Sciences & Peking Union Medical College, Tianjin, China.

5 Department of Molecular Imaging and Nuclear Medicine, Tianjin Medical University Cancer Institute and Hospital, Tianjin's Clinical Research Center for Cancer, Tianjin, China.

[#]These authors contributed equally to this work.

*Correspondences to:

Dr. Xiangqian Zheng, Huanhuxi Road, Ti-Yuan-Bei, Hexi District, Tianjin 300060, P. R. China (xzheng05@tmu.edu.cn).

Dr. Xianhui Ruan, Huanhuxi Road, Ti-Yuan-Bei, Hexi District, Tianjin 300060, P. R. China (tjruanxianhui@163.com).

Dr. Qiman Dong, Huanhuxi Road, Ti-Yuan-Bei, Hexi District, Tianjin 300060, P. R. China (18330225980@163.com).

**Table of contents**

Supplementary Figures and Supplementary Figure legends ------------------------------------ 3

Additional File 1 ------------------------------------------------------------------------------------------ 14

Additional File 2 ------------------------------------------------------------------------------------------ 18

## Supplementary Figures and Supplementary Figure legends

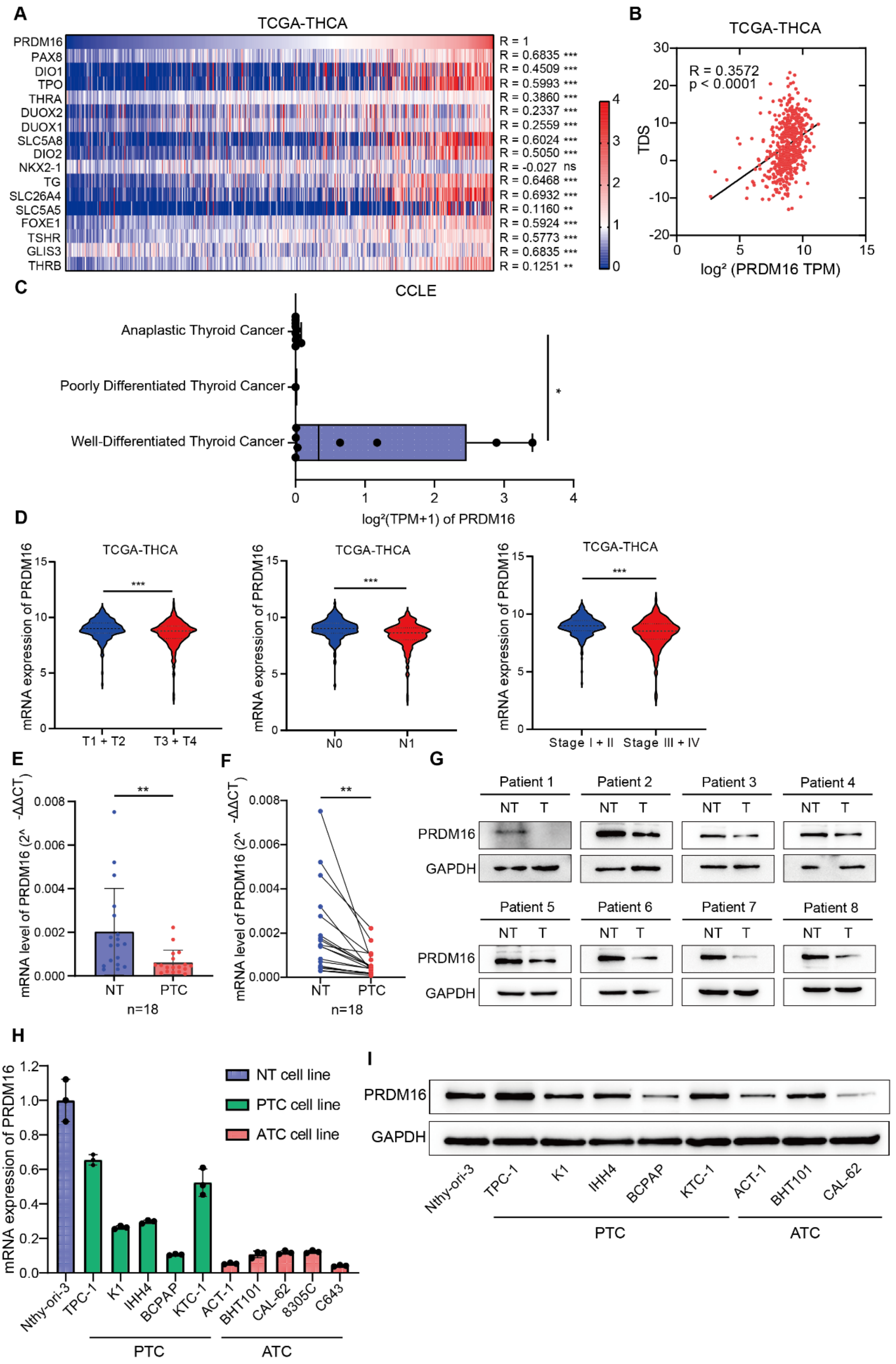


**Figure S1.** (A) Heat map illustrating the correlation between PRDM16 expression and

TDS-related genes in the TCGA thyroid cancer dataset. (B) Pearson's correlation analysis of PRDM16 expression and TDS levels in the TCGA database. (C) Comparison of PRDM16 expression among thyroid cancer subtypes in the CCLE dataset. (D) Correlation analysis of PRDM16 expression with tumor size, lymph node metastasis, and clinical stage in PTC patients from the TCGA database. (E, F) RT-qPCR analysis of PRDM16 mRNA levels in NT and PTC tissues. (G) Western blotting analysis of PRDM16 protein levels in NT and PTC tissues. (H) RT-qPCR analysis of PRDM16 mRNA levels in thyroid cancer cell lines and the normal thyroid cell line. (I) Western blotting analysis of PRDM16 protein expression in thyroid cancer cell lines and the normal thyroid cell line. All data represent mean ± SD. Statistical significance was determined by unpaired Student's t-test (C-E), paired Student's t-test (F), or Pearson′ s correlation (B).

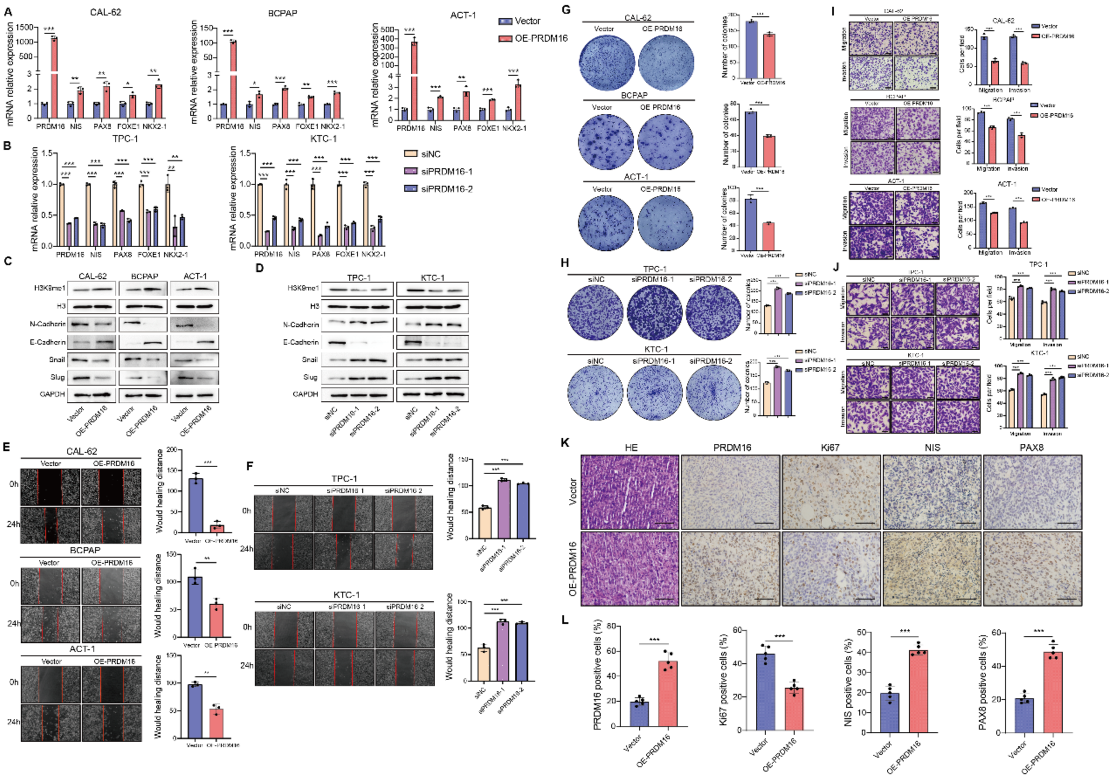

**Figure S2.** (A, B) RT-qPCR analysis detecting the mRNA expression levels of PRDM16 and thyroid differentiation markers (PAX8, NIS, TG, TPO) in thyroid cancer cells following

PRDM16 overexpression (A) or knockdown (B). (C, D) Western blotting analysis of H3K9me1, H3, and EMT-related proteins expression in thyroid cancer cells with PRDM16 overexpression (C) or knockdown (D). (E, F) Wound healing assays assessing the effects of PRDM16 overexpression (E) or knockdown (F) on the migration capability of thyroid cancer cells. (G, H) Colony formation assays of thyroid cancer cells with PRDM16 overexpression (G) or PRDM16 knockdown (H). (I, J) Transwell assays detecting the effects of PRDM16 overexpression (I) or knockdown (J) on the migration and invasion of thyroid cancer cells. Scale bar = 200 μm. (K) IHC staining showing the expression of PRDM16, Ki67, PAX8, and NIS in xenograft tumors derived from CAL-62 cells overexpressing PRDM16. Scale bar = 50 μm. (L) Quantitative analysis of immunohistochemical staining for PRDM16, Ki67, NIS, and PAX8 in xenograft tumors derived from CAL-62 cells. All data represent mean ± SD. Statistical significance was determined by unpaired Student's t-test (A, B, E-J and L).

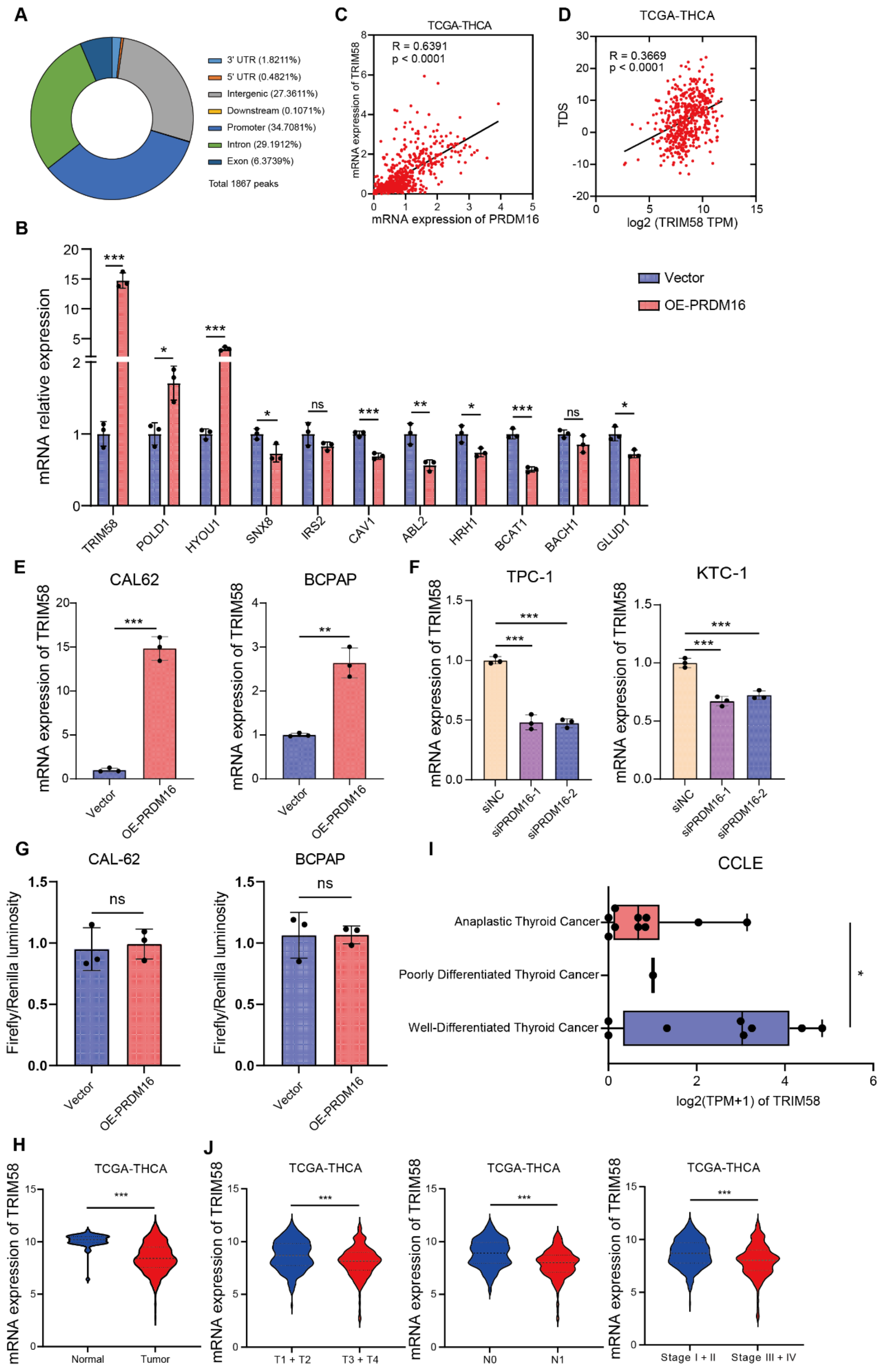


**Figure S3.** (A) Genome-wide distribution of 1,867 PRDM16-binding sites identified by

ChIP-seq using anti-Flag antibody. (B) mRNA expression of candidate genes in PRDM16-overexpressing thyroid cancer cells. (C) Pearson's correlation analysis of TRIM58 expression and PRDM16 expression in the TCGA database. (D) Pearson's correlation analysis of TRIM58 expression and TDS levels in the TCGA database. (E, F) RT-qPCR analysis of TRIM58 mRNA expression in thyroid cancer cells following PRDM16 overexpression (E) or knockdown (F). (G) Dual-luciferase assay for the transcriptional regulation of TRIM58 promoter by PRDM16. (H) Analysis of TRIM58 mRNA expression in papillary thyroid carcinoma and normal thyroid tissues in the TCGA dataset. (I) PRDM16 expression levels across thyroid cancer subtypes in the CCLE dataset. (J) Correlation analysis of TRIM58 expression with tumor size, lymph node metastasis, and clinical stage in PTC patients from the TCGA database. All data represent mean ± SD. Statistical significance was determined by unpaired Student's t-test (B-J) or Pearson's correlation (C, D).

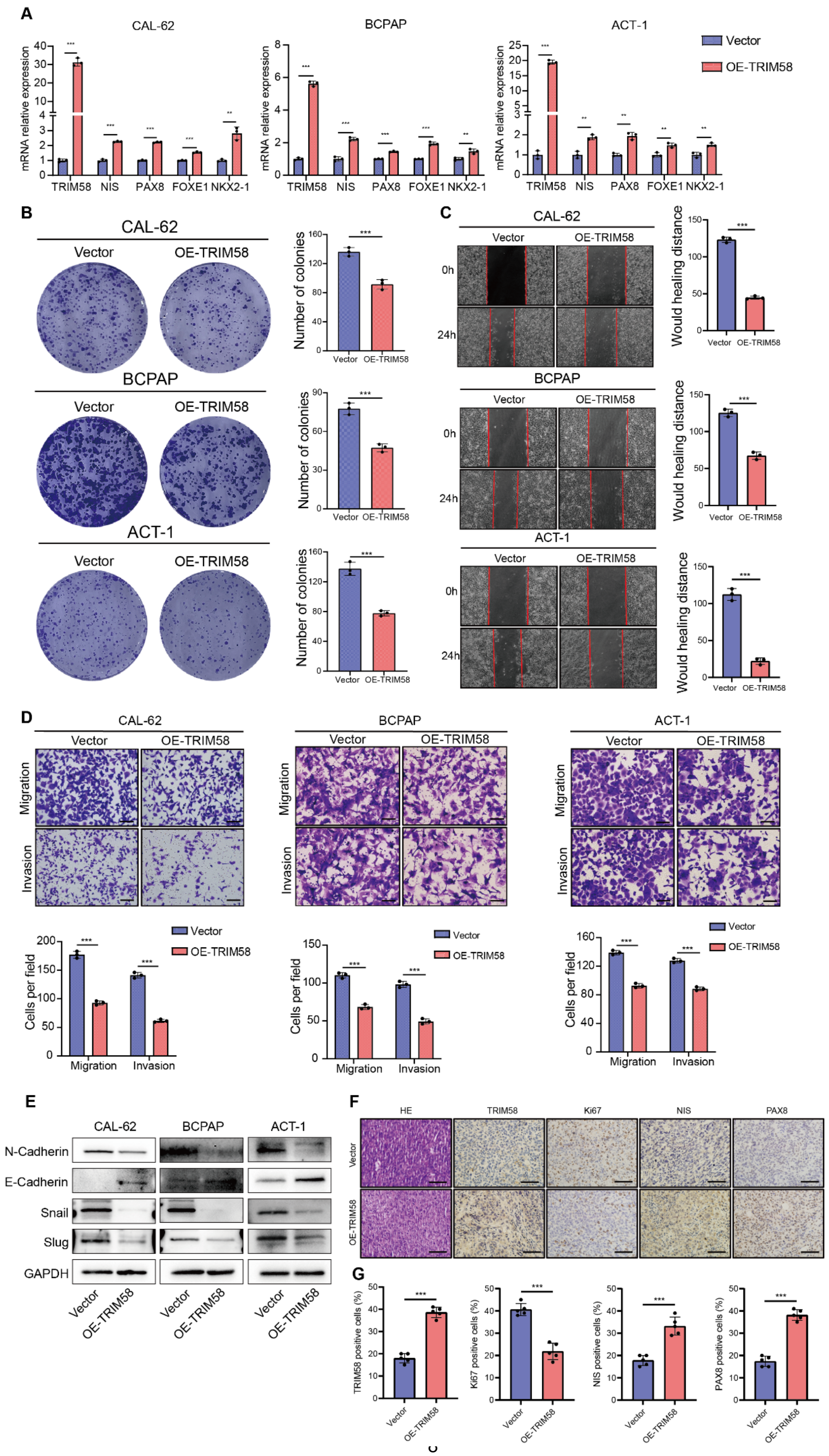
A
CAL-62
BCPAP
ACT-1
Vector
OE-TRIM58
mRNA relative expression
TRIM58 NIS PAX8 FOXE1 NKX2-1
B
Number of colonies
C
0h
24h
Would healing distance
D
Migration
Invasion
Cells per field
E
N-Cadherin
E-Cadherin
Snail
Slug
GAPDH
F
HE
TRIM58
Ki67
NIS
PAX8
G
TRIM58 positive cells (%)
Ki67 positive cells (%)
NIS positive cells (%)
PAX8 positive cells (%)

**Figure S4.** (A) RT-qPCR analysis detecting the mRNA expression of TRIM58 and thyroid differentiation markers in thyroid cancer cells following TRIM58 overexpression. (B) Colony formation assay of thyroid cancer cells overexpressing TRIM58. (C) Wound healing assay evaluating the effects of TRIM58 overexpression on the migration ability of thyroid cancer cells. (D) Transwell assay determining the cell migration and invasion abilities of thyroid cancer cells following TRIM58 overexpression. Scale bars = 200 μm. (E) Western blotting analysis of EMT-related proteins expression in thyroid cancer cells overexpressing TRIM58. (F) IHC staining showing the expression of TRIM58, Ki67, PAX8, and NIS in xenograft tumors derived from CAL-62 cells with TRIM58 overexpression. Scale bar = 50 μm. (G) Quantitative analysis of IHC staining for TRIM58, Ki67, NIS, and PAX8 in xenograft tumors derived from CAL-62 cells overexpressing TRIM58. All data represent mean ± SD. Statistical significance was determined by unpaired Student's t-test (A-D, and G).

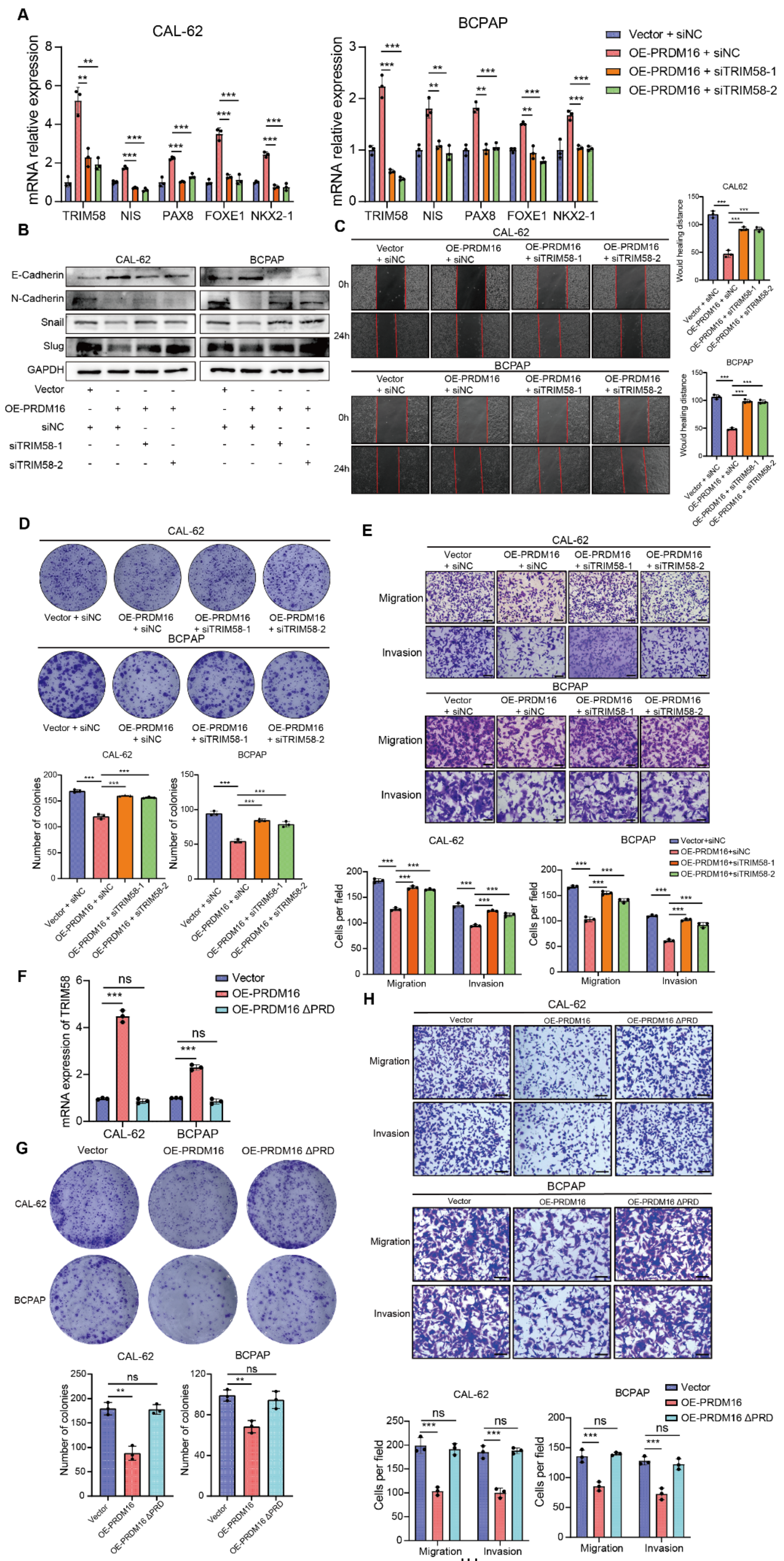
A
CAL-62
BCPAP
mRNA relative expression
TRIM58
NIS
PAX8
FOXE1
NKX2-1
Vector + siNC
OE-PRDM16 + siNC
OE-PRDM16 + siTRIM58-1
OE-PRDM16 + siTRIM58-2
B
E-Cadherin
N-Cadherin
Snail
Slug
GAPDH
Vector
OE-PRDM16
siNC
siTRIM58-1
siTRIM58-2
C
0h
24h
Would healing distance
CAL62
D
Number of colonies
E
Migration
Invasion
Cells per field
Vector+siNC
OE-PRDM16+siNC
OE-PRDM16+siTRIM58-1
OE-PRDM16+siTRIM58-2
F
mRNA expression of TRIM58
Vector
OE-PRDM16
OE-PRDM16 ΔPRD
G
H

**Figure S5.** (A) RT-qPCR analysis of thyroid differentiation marker expression levels in PRDM16-overexpressing cells with or without TRIM58 knockdown. (B) Western blotting analysis of EMT-related protein expression in PRDM16-overexpressing cells with or without TRIM58 knockdown. (C) Wound healing assay evaluating the migration ability of PRDM16-overexpressing cells with or without TRIM58 knockdown. (D) Colony formation assay assessing the influence of TRIM58 knockdown on the proliferative capacity of PRDM16-overexpressing thyroid cancer cells. (E) Transwell assays detecting the effects of TRIM58 knockdown on the migration and invasion abilities of PRDM16-overexpressing thyroid cancer cells. Scale bar = 200 μm. (F) RT-qPCR analysis of TRIM58 mRNA expression in cells overexpressing wild-type PRDM16 or PR domain–deficient PRDM16. (G) Colony formation assay of thyroid cancer cells overexpressing wild-type PRDM16 or PR domain–deficient PRDM16. (H) Transwell assay evaluating the migration and invasion abilities of thyroid cancer cells overexpressing wild-type PRDM16 or PR domain–deficient PRDM16. All data represent mean ± SD. Statistical significance was determined by unpaired Student's t-test (A, C-H).

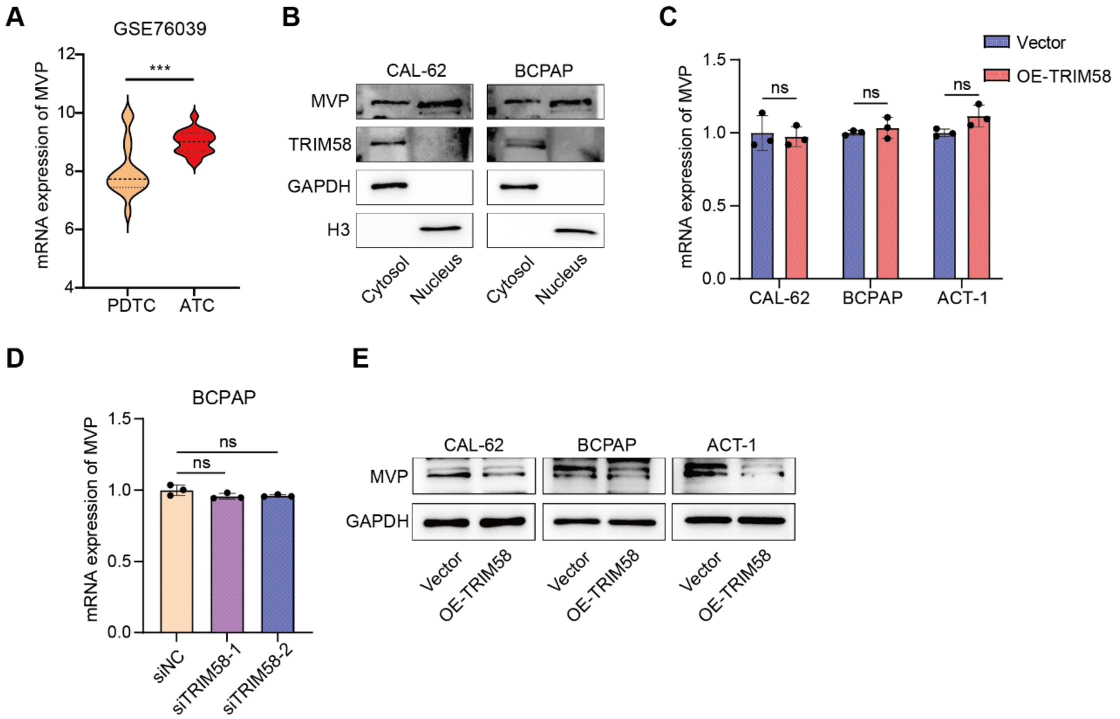


**Figure S6.** (A) Analysis of MVP expression in different types of thyroid carcinoma and normal thyroid tissue in the GSE76039 database. (B) Subcellular localization of MVP and TRIM58 proteins in thyroid cancer cell lines determined by nuclear-cytoplasmic fractionation. (C, D) RT-qPCR analysis of MVP mRNA expression in thyroid cancer cells with TRIM58 overexpression (C) or knockdown (D). (E) Western blotting analysis of MVP expression in thyroid cancer cells with TRIM58 overexpression. All data represent mean ± SD. Statistical significance was determined by unpaired Student's t-test (A-C).

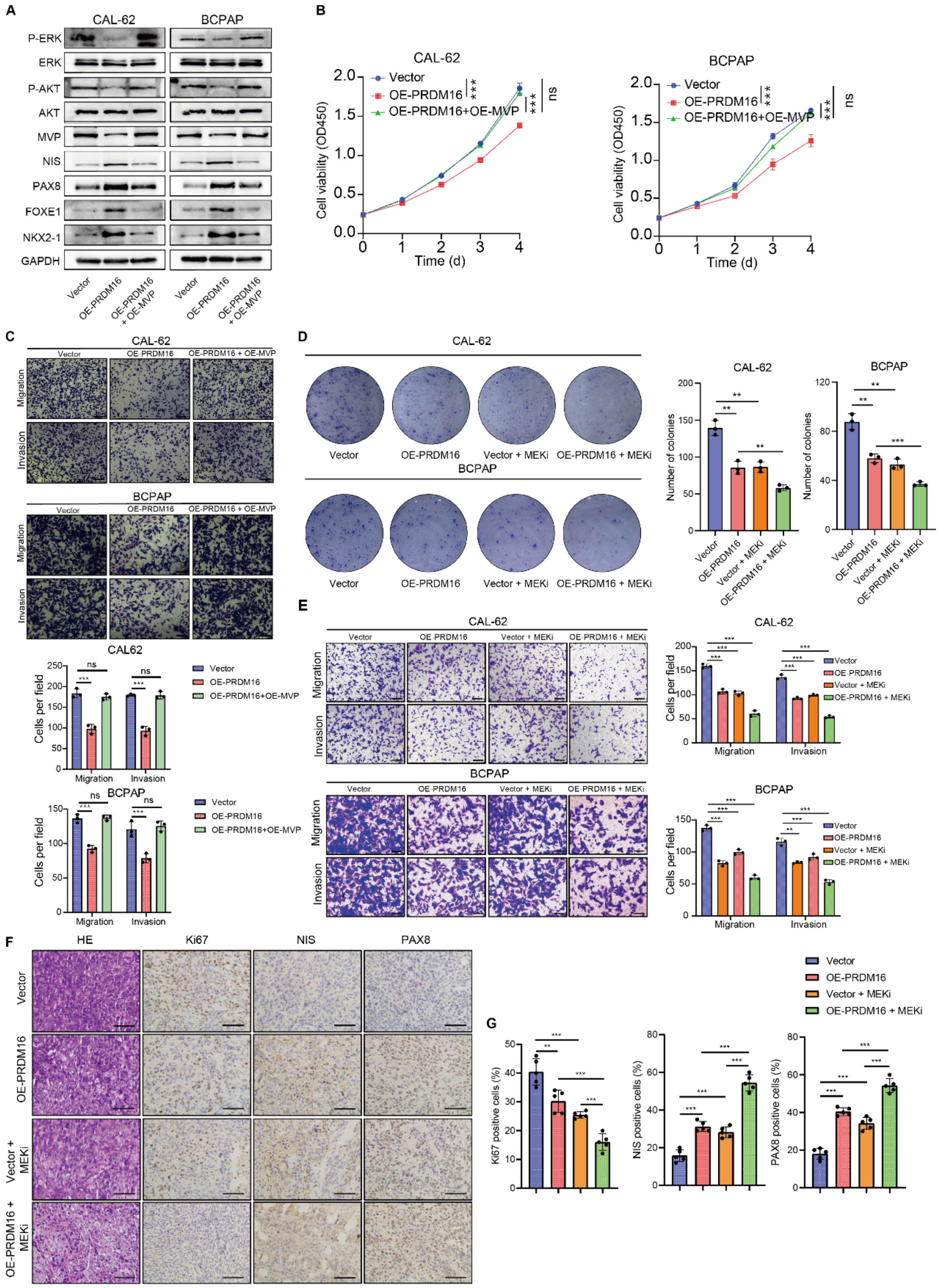

**Figure S7.** (A) Western blotting analysis of MAPK and PI3K/AKT pathway activity and thyroid differentiation markers in PRDM16-overexpressing cells with or without MVP overexpression. (B) CCK-8 assays showing the effects of MVP overexpression on cell

proliferation in PRDM16-overexpressing thyroid cancer cells. (C) Transwell assays detecting the effects of MVP overexpression on the migration and invasion abilities of PRDM16-overexpressing thyroid cancer cells. Scale bar = 200 μm. (D) Colony formation assay of thyroid cancer cells with PRDM16 overexpression, with or without 1 μM selumetinib treatment. (E) Transwell assays showing the effects of 1 μM selumetinib treatment on the migration and invasion abilities of PRDM16-overexpressing thyroid cancer cells. (F) IHC staining was performed to evaluate the expression levels of Ki67, PAX8, and NIS in xenografts generated from CAL-62 cells with or without PRDM16 overexpression following 1 μM selumetinib treatment. Scale bar = 50 μm. (G) Quantitative analysis of IHC staining results for Ki67, NIS, and PAX8. All data represent mean ± SD. Statistical significance was determined by unpaired Student's t-test (B-E, and G).

**Additional File 1**

1.Primer for RT-qPCR

| Name | Forward Primer (5`-3`) | Reverse Primer (5`-3`) |
|---|---|---|
| β-actin | TGACGTGGACATCCGCAAAG | CTCCTTAATGTCACGCACGAT |
| PRDM16 | CTTTCAAGTGCCACCTGTGC | TTCTCGTGCTCGTGCTTCTT |
| NIS | GTTCTACACTGACTGCGACCCTC | GCAGCCGAGGTTTGATGAG |
| PAX8 | AAGGTGGTGGAGAAGATTGG | AGGGAGGTTGAATGGTTGC |
| NKX2-1 | GTACCAGGACACCATGAGGAAC | CCATGTTCTTGCTCACGTC |
| FOXE1 | CACGGTGGACTTCTACGGG | GGACACGAACCGATCTATCCC |
| TRIM58 | TACCAGGTAAAGCTCCAGATGG | GAAAGCCACGATGCTTCTCAA |

| | | |
|---|---|---|
| POLD1 | ATCCAGAACTTCGACCTTCCG | ACGGCATTGAGCGTGTAGG |
| HYOU1 | GAGGAGGCGAGTCTGTTGG | GCACTCCAGGTTTGACAATGG |
| SNX8 | TACAGACGGTACAATGACTTCGT | GCCTCGATGAACTCCCTGTC |
| IRS2 | CGGTGAGTTCTACGGGTACAT | TCAGGGTGTATTCATCCAGCG |
| CAV1 | GCGACCCTAAACACCTCAAC | ATGCCGTCAAAACTGTGTGTC |
| ABL2 | GTTGAACCCCAGGCACTAAAT | CAACGAAGAGATTAGGGTCACTC |
| HRH1 | AGATGTGTGAGGGCAACAAGA | CAAGCAGATAGTGCTCAGGAC |
| BCAT1 | GTGGAGTGGTCCTCAGAGTTT | AGCCAGGGTGCAATGACAG |
| BACH1 | TCTGAGTGAGAACTCGGTTTTTG | CGCTGGTCATTAAGGCTGAGTAA |
| GLUD1 | GGAGGAGTGACAGTATCTTAC | GTATGCCAAGCCAGAGTG |
| MVP | TACATCCGGCAGGACAATGAG | CTGTGCAGTAGTGACGTGGG |

2. Primers for ChIP-qPCR

| Name | Forward Primer (5`-3`) | Reverse Primer (5`-3`) |
|---|---|---|
| TRIM58-promoter-P1 | TGACAGGTACACCACTGAGAGG | AGCCAGTCTTGGAGATGACAAAG |
| TRIM58-promoter-P2 | CGGGAGGCGGGTCATGG | GCAGGAAATCCAGGCACACC |
| TRIM58-promoter-P3 | TGTCTTAACCAGCCAGGAAACC | GTCAGCAAAAGCAAGGAGATGTAG |

3. Sequences of siRNA

| Name | Sequence (5`-3`) | Sequence (3`-5`) |
|---|---|---|

| | | |
|---|---|---|
| siNC | UUCUCCGAACGUGUCACGUTT | ACGUGACACGUUCGGAGAATT |
| siPRDM16-1 | GCCUGUGUGUGCGCCACAATT | UUGUGGCGCACACACAGGCTT |
| siPRDM16-2 | CAAGCUCUGCGGAAAUCAATT | UUGAUUUCCGCAGAGCUUGTT |
| siTRIM58-1 | GCUUCAGAUUGGAGUUUGATT | UCAAACUCCAAUCUGAAGCTT |
| siTRIM58-2 | CAAUAUCCUUGUAUUUCAATT | UUGAAAUACAAGGAUAUUGTT |

4. Antibodies and drugs

| Antibodies name | Application | Supplier | Cat # |
|---|---|---|---|
| Anti-human GAPDH antibody | WB | ABclonal | AC002 |
| Anti-human PRDM16 antibody | WB, IHC | Proteintech | 83872-1-RR |
| Anti-human NIS antibody | WB, IHC | Proteintech | 24324-1-AP |
| Anti-human PAX8 antibody | WB, IHC | BOSTER | A00943-4 |
| Anti-human NKX2-1 antibody | WB | Bimake | A5549 |
| Anti-human FOXE1 antibody | WB | Santa Cruz | sc-518211 |
| Anti-human E-Cadherin antibody | WB | Proteintech | 20874-1-AP |
| Anti-human N-Cadherin antibody | WB | Proteintech | 22018-1-AP |
| Anti-human Snail antibody | WB | Cell Signaling Technology | 3879 |
| Anti-human Slug antibody | WB | Cell Signaling Technology | 9585 |
| Anti-human H3K9me1 antibody | WB, ChIP | Abclonal | A20734 |
| Anti-human H3 antibody | WB | Abclonal | A2348 |

| | | | |
|---|---|---|---|
| Anti-human TRIM58 antibody | WB, IHC | Sigma-Aldrich | HPA023637 |
| Anti-human MVP antibody | WB | Proteintech | 16478-1-AP |
| Anti-human MEK antibody | WB | Cell Signaling Technology | 8727 |
| Anti-human P-MEK antibody | WB | Cell Signaling Technology | 9154 |
| Anti-human ERK antibody | WB | Cell Signaling Technology | 4695 |
| Anti-human P-ERK antibody | WB | Cell Signaling Technology | 4370 |
| Anti-human AKT antibody | WB | Abclonal | A18675 |
| Anti-human P-AKT antibody | WB | Abclonal | AP0637 |
| Anti-human Ki67 antibody | IHC | Proteintech | 27309-1-AP |
| Anti Flag-tag antibody | WB, ChIP, IP | Cell Signaling Technology | 14793 |
| Anti Myc-tag antibody | WB, IP | Proteintech | 16286-1-AP |
| Anti ubiquitin antibody | WB | Proteintech | 10201-2-AP |
| MG132 | / | Selleck | S2619 |
| CHX | / | Selleck | S7418 |
| Selumetinib | / | Selleck | S1008 |

**Additional file 2**

**Cell Lines and Cell Culture**

The Nthy-ori-3-1, TPC-1, K1, IHH4, BCPAP, KTC-1, ACT-1, BHT101, CAL-62, 8305C, and C643 cell lines used in this study were obtained from the American Type Culture Collection (ATCC). All cell lines were authenticated by short tandem repeat (STR) analysis. Cells were cultured in Dulbecco's modified Eagle's medium (DMEM, Gibco) supplemented with 10% fetal bovine serum (FBS) and maintained at 37 °C in a humidified incubator with 5% $CO_2$.

**Cell Viability Assay and Colony Formation Assay**

Cells were seeded in 96-well culture plates at 2000 cells per well. Cell viability was measured using a Cell Counting Kit-8 (CCK-8) (SolarBio, China) according to the manufacturer's instructions. The colony formation assay was employed to assess cellular proliferative capacity. Cells were seeded in 6-well plates at a density of 2000 cells per well and cultured for approximately 10-14 days until macroscopic colonies became clearly visible.

**Transwell Assay and Wound Healing Assay**

Cell migration assays were performed using polycarbonate membrane Transwell chambers (8 μm pore size; Corning, USA) in 24-well plates. DMEM basal medium (500 μL) containing 20% FBS was added to the lower chamber. The 200 μL cell suspension (1 ×

$10^6$ cells/mL) in serum-free medium was added and incubated in the upper chamber at 37 °C with 5% $CO_2$ for 24 h. The Transwell chamber was fixed in 5% glutaraldehyde and stained with 0.1% crystal violet. For the invasion assay, the Transwell chamber was coated with Matrigel (BD Biosciences, USA) before adding the cell suspension. For the wound healing assay, cells were plated to confluence in a 6-well plate and the cell monolayer was scratched with a pipette tip. Wound healing within the scratch line was observed at the indicated time points and representative scratch lines for each cell line were photographed.

**Dual-luciferase Assay**

Cells were seeded into 24-well plates and cultured until they reached 70%–80% confluence. Transfection mixtures were prepared and applied to the cells, with each complex consisting of 0.5 μg of reporter plasmid, 0.05 μg of Renilla plasmid, 0.5 μg of expression plasmid, and Lipofectamine 3000. After 48 h of incubation, the dual-luciferase assay was performed according to the manufacturer's protocol (Promega). The relative reporter activity was calculated as the ratio of firefly luciferase activity to Renilla luciferase activity (Firefly/Renilla).

**Immunofluorescence (IF)**

Cells were fixed with 4.0% paraformaldehyde for 15 min, permeabilized with 0.3% Triton X-100 for 10 min and blocked with 5% goat serum for 30 min. After blocking, cells were incubated overnight at 4 °C with primary antibodies. The next day, cells were incubated with fluorescently conjugated secondary antibodies, counterstained with DAPI (4′,6-diamidino-2-phenylindole), and imaged using a fluorescence microscope.

**Nuclear-Cytoplasmic Protein Fractionation**

Nuclear and cytoplasmic proteins were extracted from thyroid cancer cells using the EK-6100 Nuclear and Cytoplasmic Protein Extraction Kit (ECOTOP SCIENTIFIC) according to the manufacturer's protocol. After fractionation, nuclear and cytoplasmic proteins were subjected to SDS-PAGE followed by Western blot analysis to assess protein distribution in the nuclear and cytoplasmic fractions. Histone H3 and GAPDH were used as nuclear and cytoplasmic marker proteins, respectively, to evaluate the purity of the fractionation.
